# Ledipasvir plus sofosbuvir in pregnant women with hepatitis C virus infection: a phase 1 pharmacokinetic study

**DOI:** 10.1016/S2666-5247(20)30062-8

**Published:** 2020-07-27

**Authors:** Catherine A Chappell, Kimberly K Scarsi, Brian J Kirby, Vithika Suri, Anuj Gaggar, Debra L Bogen, Ingrid S Macio, Leslie A Meyn, Katherine E Bunge, Elizabeth E Krans, Sharon L Hillier

**Affiliations:** Department of Obstetrics, Gynecology, and Reproductive Sciences, University of Pittsburgh, Pittsburgh, PA, USA; Magee-Womens Research Institute, Pittsburgh, PA, USA; College of Pharmacy, University of Nebraska Medical Center, Omaha, NE, USA; Gilead Sciences, Foster City, CA, USA; Gilead Sciences, Foster City, CA, USA; Gilead Sciences, Foster City, CA, USA; Department of Pediatrics, University of Pittsburgh, Pittsburgh, PA, USA; Magee-Womens Research Institute, Pittsburgh, PA, USA; Magee-Womens Research Institute, Pittsburgh, PA, USA; Department of Obstetrics, Gynecology, and Reproductive Sciences, University of Pittsburgh, Pittsburgh, PA, USA; Magee-Womens Research Institute, Pittsburgh, PA, USA; Department of Obstetrics, Gynecology, and Reproductive Sciences, University of Pittsburgh, Pittsburgh, PA, USA; Magee-Womens Research Institute, Pittsburgh, PA, USA; Department of Obstetrics, Gynecology, and Reproductive Sciences, University of Pittsburgh, Pittsburgh, PA, USA; Magee-Womens Research Institute, Pittsburgh, PA, USA

## Abstract

**Background:**

Hepatitis C virus (HCV) infection is increasing among pregnant women because of the opioid epidemic, yet there are no interventions to reduce perinatal HCV transmission or to treat HCV during pregnancy. Physiological changes in pregnancy alter the pharmacokinetics of some medications; thus, our aim was to compare the pharmacokinetic parameters of ledipasvir 90 mg plus sofosbuvir 400 mg during pregnancy with non-pregnant women.

**Methods:**

This was an open-label, phase 1 study of pregnant women with genotype 1 HCV infection and their infants. A reference group of women who had participated in pharmacokinetic studies of ledipasvir–sofosbuvir during phase 2 and 3 trials was used. Participants were enrolled at Magee-Womens Hospital (Pittsburgh, PA, USA) between 23 and 24 weeks’ gestation and had a 12-week course of oral ledipasvir–sofosbuvir (daily 90 mg ledipasvir plus 400 mg sofosbuvir). Three 12-h intensive pharmacokinetic visits were done at 25–26, 29–30, and 33–34 weeks’ gestation and individual pharmacokinetics were summarised by geometric mean across the three visits. The primary outcome, analysed in all participants without suspected dosing errors, was the ledipasvir–sofosbuvir area under the concentration–time curve of the dosing interval (AUC_tau_) during pregnancy compared with the reference group by geometric mean ratio. This study is registered with ClinicalTrials.gov, NCT02683005.

**Findings:**

From Oct 1, 2016, to Sept 30, 2018, 29 pregnant women were screened and nine (31%) were enrolled. Eight (89%) women were included in the primary analysis. Ledipasvir and sofosbuvir exposures were similar in the pregnant women versus the non-pregnant reference group (geometric mean ratio of AUC_tau_ ledipasvir 89·3% [90% CI 68·7–116·1]; sofosbuvir 91·1% [78·0–106·3]).

**Interpretation:**

Ledipasvir–sofosbuvir was safe and effective without clinically meaningful differences in drug exposure among pregnant versus non-pregnant women.

**Funding:**

National Institutes of Health/Eunice Kennedy Shriver National Institute of Child Health and Human Development, the National Institutes of Health/Office of Research on Women’s Health, and Gilead Sciences.

## Introduction

In 2015, WHO estimated that 71·1 million people were infected with hepatitis C virus (HCV), with 1·75 million new infections.^1^ Injection drug use among people of reproductive age is the leading cause of new HCV infections^2^ and has resulted in a dramatic rise in the prevalence of HCV among pregnant women.^3,4^ From 2006 to 2012, the prevalence of HCV among young people increased by 364% in areas of the USA that were the hardest hit by the opioid epidemic, including Kentucky, Tennessee, Virginia, and West Virginia.^5^ Although the rate of perinatal HCV transmission ranges from 4% to 8% and is twice as high among individuals with HIV co-infection,^6^ there are no proven interventions to decrease the risk of perinatal transmission and neonatal infection.^7,8^ Pregnancy is a crucial period of health-care engagement, and HCV treatment during pregnancy would not only cure maternal HCV infection, but might also prevent perinatal HCV transmission and future community HCV transmission. In a retrospective cohort study of 369 women with opioid use disorder and HCV infection, only six women received HCV treatment in the year following delivery, showing that there are barriers to HCV treatment provision in the post-partum period.^9^

Historically, the treatment of HCV during pregnancy was not possible because treatment included pegylated interferon and ribavirin, which have high toxicity, substantial side-effects, and teratogenicity risks. The October, 2014, US Food and Drug Administration (FDA) and November, 2014, European Medicines Agency approvals of the fixed dose combination of the NS5B polymerase inhibitor sofosbuvir and the NS5A inhibitor ledipasvir for the treatment of treatment-naive and treatment-experienced HCV genotype 1a and 1b infection has marked a new era of HCV treatment, with a more than 95% cure rate for patients without cirrhosis.^10^ Pregnant women with HCV do not receive treatment during pregnancy because there is an absence of data regarding the safety and efficacy of highly effective direct-acting antivirals in pregnancy. Pregnant women are usually excluded from participating in clinical trials of new drugs. Prospective evaluations of medications for use in pregnancy often lag by years after approval or are not done at all.^11^ A fundamental first step towards the development of a clinical pathway for treating HCV during pregnancy is to establish safe and therapeutic drug exposures during pregnancy.

Ledipasvir–sofosbuvir is a candidate for treatment of HCV during pregnancy because preclinical data showed no safety concerns when ledipasvir and sofosbuvir were administered to pregnant rats and rabbits at higher doses than those used to treat HCV in humans.^12^ On the basis of drug interaction studies, ledipasvir bioavailability in humans is estimated to be less than 30%.^13^ Ledipasvir requires an acidic environment for absorption, is 99·8% protein bound, 30% is hepatically metabolised by unknown oxidative pathways, and it is primarily eliminated via biliary excretion, resulting in a plasma half-life of 47 h.^12^ Sofosbuvir is a phosphoramidate prodrug and is hydrolysed intracellularly by human catepsin A or carboxylesterase 1 and nucleotide-binding protein 1 to the pharmacologically active intracellular GS-331007 triphosphate, which is dephosphorylated to the primary circulating metabolite GS-331007. The bioavailability of sofosbuvir is more than 80% and a high-fat meal increases sofosbuvir exposure by 67–91%. Sofosbuvir is 61–65% plasma protein bound, 14% is hepatically metabolised by phosphorylation, and 80% is renally excreted. As a prodrug, the sofosbuvir half-life is 0·4 h, and the half-life of GS-331007 is 27 h.^14^ Given the substantial physiological changes in pregnancy that influence the pharmacokinetics of many medications,^15^ the primary objective of this study was to compare the pharmacokinetic parameters of ledipasvir–sofosbuvir administered to pregnant women with HCV infection to those of a historical reference group of non-pregnant women with HCV infection. The secondary objectives were to define the safety of, and virological response to, ledipasvir–sofosbuvir treatment during pregnancy.

## Methods

### Study design and participants

This study was an open-label, phase 1, pharmacokinetic evaluation of ledipasvir–sofosbuvir administration during pregnancy. The study was done in a large, tertiary-care maternity hospital (Magee-Womens Hospital) in Pittsburgh, PA, USA with approximately 10 000 deliveries each year. Participants were recruited using print advertisements and by referral from prenatal or substance use treatment providers. Participants were eligible if they were between 18 and 39 years of age and between 23 and 24 weeks of gestation at the time of enrolment; had chronic HCV genotype 1, 4, 5, or 6 infection (HCV antibody detected at least 6 months before screening and detectable HCV RNA viral load at screening); and had a singleton gestation with no known fetal abnormalities. Participants were excluded if they had HIV or hepatitis B co-infection, previous HCV treatment with sofosbuvir or an NS5A inhibitor, or a history of cirrhosis. The full inclusion and exclusion criteria are in the appendix (pp 21–22). All participants gave written informed consent before the screening procedures.

The reference group comprised women who had previously participated in intensive pharmacokinetic studies of ledipasvir–sofosbuvir during registrational phase 2 and 3 clinical trials. Data for the reference group were provided by Gilead Sciences. Because age, sex, race, and bodyweight have not been found to affect ledipasvir–sofosbuvir pharmacokinetics, the reference group was used without stratification.^13^

This study was done under an FDA investigational new drug application (number 129502) from the investigator at the University of Pittsburgh. Recruitment materials and the study protocol (appendix pp 1–56) were approved by the University of Pittsburgh Institutional Review Board.

### Procedures

Participants initiated a 12-week course of once daily ledipasvir 90 mg plus sofosbuvir 400 mg fixed dose combination, administered orally between 23 and 24 weeks of gestation. Although equal efficacy has been shown with an 8-week course of ledipasvir–sofosbuvir for treatment-naive patients,^16^ a more conservative 12-week course was chosen because of concerns regarding the physiological immunosuppression associated with pregnancy and that an 8-week course might not be adequate. Adherence throughout the study was assessed by pill count and participant report using a medication log. Participants returned to Magee-Womens Hospital for three 12-h intensive pharmacokinetic visits at 25–26, 29–30, and 33–34 weeks of gestation.

At each pharmacokinetic visit, participants took a timed dose of ledipasvir–sofosbuvir with a standardised breakfast with moderate fat and calories (approximately 600 kcal and 25–30% fat) following a 10-h overnight fast. Blood samples of 4 mL were collected before taking the study drug and at 30 min, and 1, 2, 3, 4, 5, 8, and 12 h after the observed dose. An additional 12 mL of blood was collected at the 2-h timepoint for protein binding analysis. Food intake was restricted until after the collection of the blood sample at 4 h. All blood samples were immediately processed and stored at −70°C. Plasma concentrations of ledipasvir, sofosbuvir, and GS-331007 were measured by validated high-performance liquid chromatography–tandem mass spectrometry methods at QPS laboratories (Neward, DE, USA).^17^ The assay range was 1–2000 ng/mL for ledipasvir, 5–2500 ng/mL for sofosbuvir, and 10–5000 ng/mL for GS-331007. The free-fraction of ledipasvir and sofosbuvir were assessed by equilibrium dialysis. The assays for ledipasvir, sofosbuvir, and GS-331007 for the reference group were done using the same method and in the same laboratory as for the participants enrolled as part of this study.

At each pharmacokinetic visit, an HCV viral load was measured and adverse events were assessed. If the participant delivered at Magee-Womens Hospital, then HCV viral load was assessed from the umbilical cord blood and the delivery outcomes were collected while the participant was in the post-partum ward. Sustained virological response 12 weeks after completion of treatment (SVR12), defined as undetectable HCV RNA, was collected 12 weeks after the last dose of ledipasvir–sofosbuvir and is indicative of HCV cure.^18^ The infants were followed up until 12 months of age, with scheduled visits at 8 weeks, 6 months, and 12 months. Infants were assessed for HCV infection with HCV RNA viral loads at 8 weeks, and 6 months, and at 12 months if earlier assessments did not document negative viral loads.

Safety laboratory tests (blood counts, liver function tests, and creatinine), maternal vital signs, fetal heart rate assessment, and physical examinations were done at screening and at each maternal follow-up visit (ie, at each pharmacokinetic visit). Vital signs, physical examinations, and infant growth (bodyweight, length, and head circumference) were assessed at each infant follow-up visit (at 8 weeks, 6 months, and 12 months). Infant neurodevelopment was assessed by the Bayley Scales of Infant and Toddler Development, third edition at the 6-month and 12-month visit and by the Ages and Stages Questionnaires, third edition at the last study contact at 12 months. Adverse events and concomitant medications were assessed at each maternal and infant follow-up visit. Adverse events were graded using the Division of AIDS toxicology grading table^19^ and relatedness to the study drug was determined by the study physician. Each month, all adverse events were reviewed by an independent maternal fetal medicine specialist and by the study oversight team. Delivery outcomes were assessed by chart review. Given the small sample size, pregnancy outcomes and adverse events were reported descriptively.

### Outcomes

The primary outcome was systemic plasma exposures of both ledipasvir and sofosbuvir (sofosbuvir and GS-0331007) at 25–26, 29–30, and 33–34 weeks of gestation. The secondary outcomes were SVR12; safety in the mother (laboratory assessments of blood counts and liver function at the first two pharmacokinetic visits); pregnancy and delivery outcomes measured prospectively; and safety in the neonate (major malformations; bodyweight, length, and head circumference at birth, 8 weeks, 6 months, and 12 months; HCV RNA viral load at birth, 1–3 months, and 6 months; HCV viral load at 12 months if negative viral loads are not documented earlier; and developmental outcomes at 6 and 12 months).

### Statistical analysis

The area under the plasma concentration–time curve of the dosing interval (AUC_tau_) was calculated by the trapezoidal rule using Phoenix WinNonLin version 7.0. Although ledipasvir and sofosbuvir were administered once daily, our intensive pharmacokinetic sampling occurred over 12 h and the pre-dose sample was imputed to infer the expected concentration 24 h after observed dosing. Participants were excluded from the pharmacokinetic analysis if a dosing error was suspected because of the presence of sofosbuvir in the plasma before the timed dose during the pharmacokinetic visit, because sofosbuvir is only detectable within 6 h after dosing.

We compared sofosbuvir, GS-331007, and ledipasvir pharmacokinetic parameters per visit between the study and reference groups using one-way ANOVA (assuming p>0·05 is significant). The geometric mean of individual participant pharmacokinetic parameters across the three visits was used to represent the per participant exposure during pregnancy.

The total plasma AUC_tau_, maximum observed concentration, and minimum observed concentration were summarised within the study group and the reference group using the arithmetic mean and coefficient of variation of the results from all three pharmacokinetic visits. We summarised the effect of pregnancy on the pharmacokinetic parameters by calculating the geometric mean ratio with 90% CI for the study group relative to the reference group. The total plasma pharmacokinetic parameters were considered similar if the 90% CI of the geometric mean ratio was between 70% and 143% (plus or minus 30%). For pharmacokinetic parameters with geometric mean ratios or 90% CIs falling outside these bounds, the clinical significance of the change was evaluated. The proportion of unbound drug was descriptively compared with that of the reference group. Secondary outcomes were assessed in all enrolled participants and are reported descriptively.

This study is registered with ClinicalTrials.gov, NCT02683005.

### Role of the funding source

Gilead Sciences provided the study drug, did the pharmacokinetic assays in their commercial laboratory, and provided the data for the historical reference group. Gilead Sciences also provided consultation on the study design, drafting of the protocol, and the manuscript. The National Institutes of Health had no role in study design, data collection, data analysis, data interpretation, or writing of the report. The corresponding author had full access to all the data in the study and had final responsibility for the decision to submit for publication.

## Results

From Oct 1, 2016, to Sept 30, 2018, 29 pregnant women were screened and nine (31%) were enrolled. Among screened women, 20 (69%) were excluded because of genotype 2 or 3 infection (ten [34%]), ongoing illicit drug use (four [14%]), declining study participation (three [10%]), intention to deliver off-site (two [7%]), and concern about possible cirrhosis because their aspartate aminotransferase to platelet ratio was more than 1 (one [3%]). All nine infants completed the study and all nine infants completed the 6-month HCV RNA assessment. All participants completed the 12-week course of study drug before delivery and completed the three pharmacokinetic visits. However, one participant was excluded from the pharmacokinetic analysis because of non-adherence with the dosing schedule, as evidenced by high sofosbuvir concentrations (179 and 196 ng/mL) at the pre-dose timepoint in two of the three visits (appendix p 57). Because of the short half-life of sofosbuvir, the presence of high sofosbuvir concentrations pre-dose suggested that the participant ingested sofosbuvir immediately before the scheduled visit.

All enrolled participants were white, with a median age of 31 years (range 25–38), and the majority (eight [89%]) were Medicaid insured. Although many participants had multiple risk factors for HCV infection, including having tattoos, condomless anal sex, and intravenous drug use, eight (89%) participants attributed their HCV infection to previous intravenous drug use, and one (11%) had HCV infection due to perinatal HCV transmission (table 1). The historical reference group comprised 43 women with a median age of 52 years (range 21–69), median bodyweight of 70 kg (47–136), 39 (91%) were white, three (7%) were Asian, and one (2%) was black. No further details on race or ethnicity were available in the original database.

**Figure 1:**
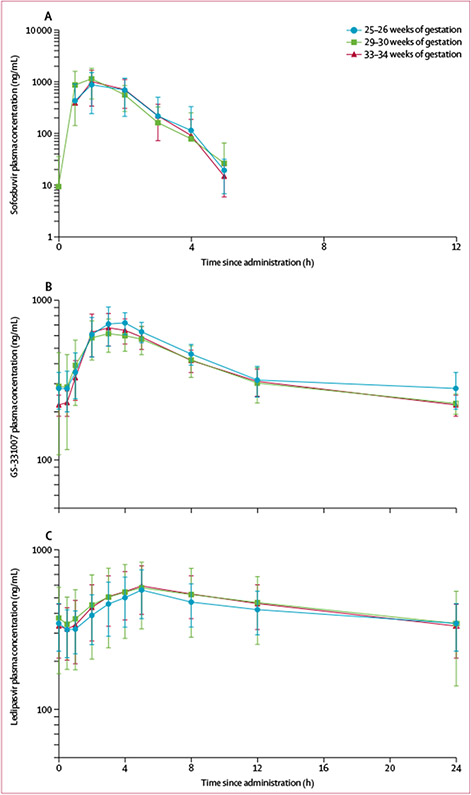
Mean concentration–time curves at each intensive pharmacokinetic visit (A) Sofosbuvir plasma profiles. (B) GS-331007 plasma profiles. (C) Ledipasvir plasma profiles. One participant was excluded from all the primary pharmacokinetic analyses because of a dosing error.

The ledipasvir, sofosbuvir, and GS-331007 pharmacokinetic parameters by gestational age are shown in table 2 and figure 1. Table 3 shows the overall pharmacokinetic parameters during pregnancy compared with the reference group. There were no clinically significant changes noted in any of the pharmacokinetic parameters for sofosbuvir or ledipasvir between pregnancy and the reference group (geometric mean ratios showed a maximum reduction of 16·5% for ledipasvir). However, GS-331007 exposure was 38% lower in pregnant women than in non-pregnant women (table 3). A post-hoc power analysis showed that eight women in the test group and 43 women in the reference group provided more than 80% power to detect a decrease in sofosbuvir or ledipasvir AUC_tau_ of more than 30%.

**Figure 2:**
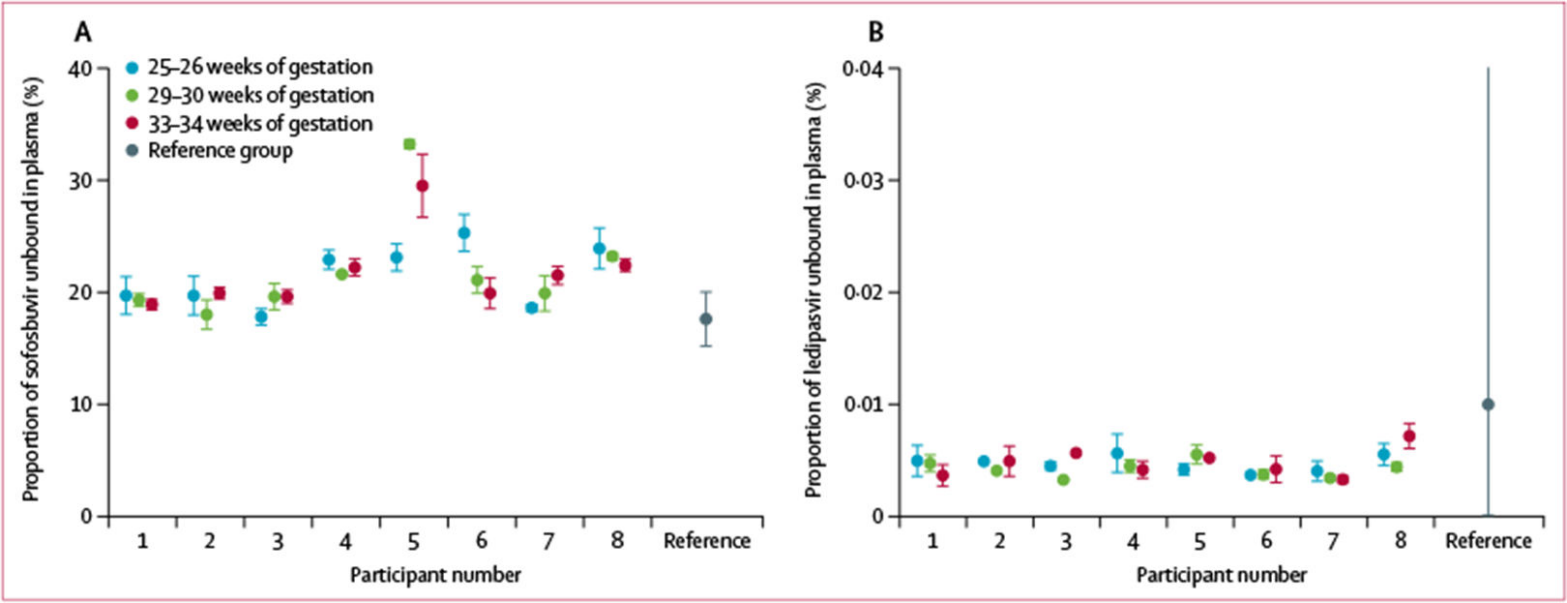
Ledipasvir and sofosbuvir plasma protein binding in the study group compared with the reference group (A) Sofosbuvir plasma protein binding. (B) Ledipasvir plasma protein binding.

Protein binding was similar across all three gestational age timepoints (figure 2). The mean proportion of sofosbuvir that was unbound during pregnancy was 21·7% (SD 3·6) compared with 17·6% (2·5) in the reference group. The mean proportion of ledipasvir that was unbound during pregnancy was 0·005% (0·001) compared with 0·014% (0·043) in the reference group (figure 2).

**Figure 3:**
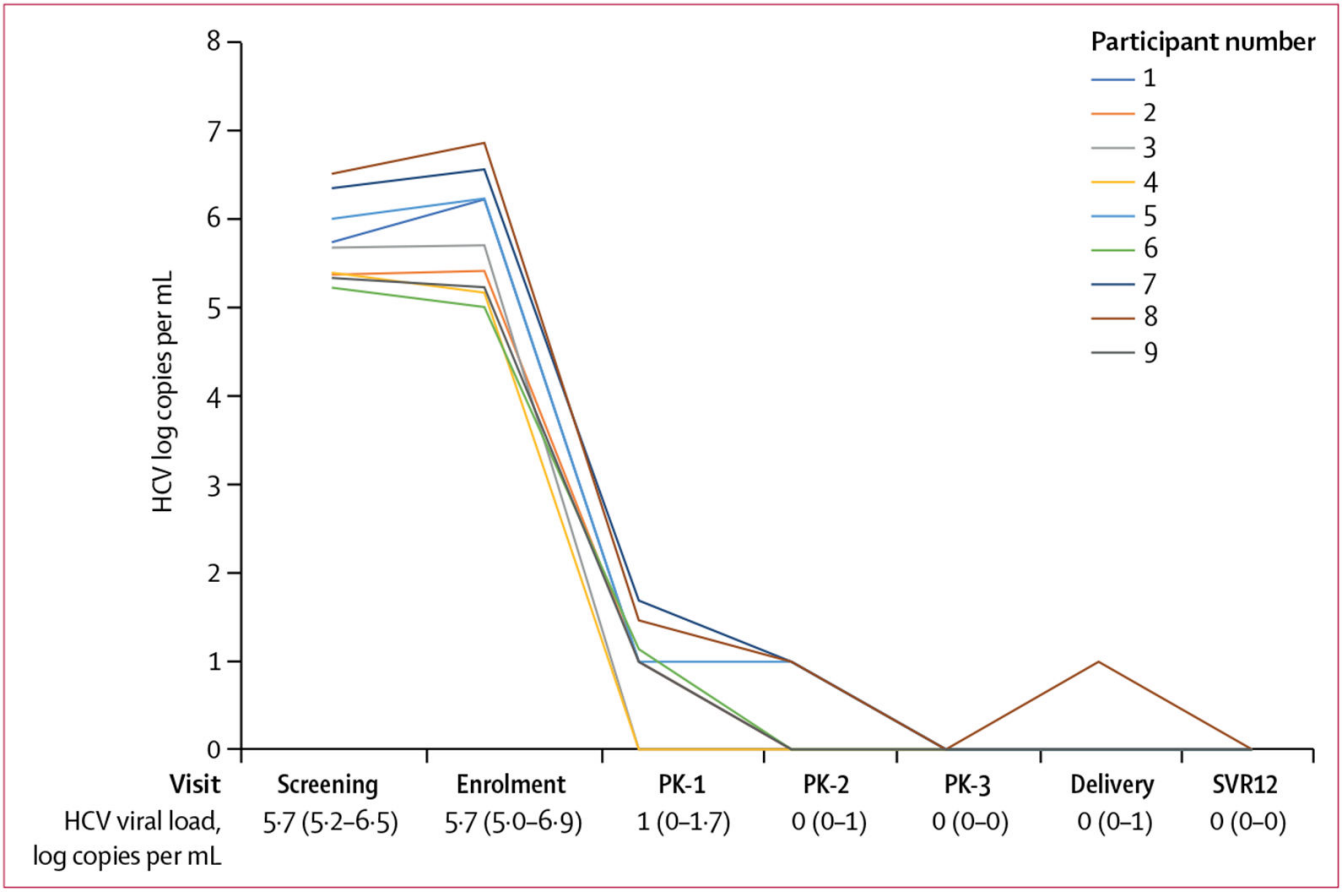
HCV viral response to ledipasvir–sofosbuvir during pregnancy Data stated below the chart are median (range). PK-1 visit was between 10 and 21 days after treatment initiation. PK-2 visit was between 32 and 47 days after treatment initiation. PK-3 visit was between 59 and 74 days after treatment initiation. HCV=hepatitis C virus. PK-1=first pharmacokinetic visit. PK-2=second pharmacokinetic visit. PK-3=third pharmacokinetic visit. SVR12=sustained virological response 12 weeks after completion of treatment.

The HCV viral load at enrolment ranged from 1·0 × 10^5^ to 73·5 × 10^5^ copies per mL, with only one participant having a viral load greater than 6 × 10^5^ copies per mL (figure 3). Upon initiation of ledipasvir–sofosbuvir, the viral load declined, and all participants had an undetectable viral load by the third pharmacokinetic visit between 33 and 35 weeks of gestation. One (11%) participant had a detectable HCV viral load at delivery; however, she also had the highest viral load at enrolment and had an opioid use relapse during the course of her treatment that resulted in approximately 15 missed doses of medication between weeks 9 and 11 of treatment. All nine (100%) participants had an undetectable viral load 12 weeks after completion of treatment, meeting the definition of HCV cure.

Five (56%) participants had an adverse event related to ledipasvir–sofosbuvir, four (44%) were grade 1 (three [33%] nausea or vomiting and one [11%] diarrhoea) and one (11%) was grade 2 (fatigue); none of these adverse events resulted in discontinuation of treatment. Eight (89%) infants delivered at term (37 weeks of gestation or more) and nine (100%) had normal birthweights more than 2500 g. The preterm infant delivered at 36 weeks and 6 days because of severe gestational hypertension. None of the infants had detectable HCV RNA at birth by cord blood sampling. Three infants were admitted to the neonatal intensive care unit for treatment for neonatal opioid withdrawal syndrome (two [22%]) and a brachial plexus injury (one [11%]) resulting from a shoulder dystocia at the time of delivery (table 4). During the 12-month follow-up, none of the infants had a detectable HCV RNA test. 60 infant adverse events were recorded during the 12-month follow-up, none of which were related to ledipasvir–sofosbuvir exposure. Of the 60 infant adverse events, 40 (67%) were grade 1, 15 (25%) were grade 2, four were grade 3 (7%), and one (<2%) was grade 4. The grade 3 and 4 adverse events included two (22%) of nine infants who required admission to hospital for treatment of neonatal opioid withdrawal syndrome, one (11%) infant who required admission to hospital for a viral illness at 7 months of age, and one (11%) infant who required admission to the neonatal intensive care unit for respiratory distress (grade 3) after a shoulder dystocia (grade 4). The infant growth parameters (bodyweight, length, head circumference) were all within the normal range and trajectory throughout the 12-month follow-up period. Two (22%) infants were referred for early intervention during the follow-up because of concerns regarding motor development; however, all nine infants had a normal neurodevelopmental assessment at the last contact. All of the infants were assessed for congenital abnormalities at birth and at each follow-up visit and none were identified. All of the maternal safety laboratory evaluations, which included a complete blood count, liver function tests, and creatinine, were normal during treatment as well as the post-treatment assessment.

## Discussion

In this phase 1 study of antenatal HCV treatment with ledipasvir–sofosbuvir, no clinically significant pharmacokinetic differences in total ledipasvir or total sofosbuvir were identified in pregnant women compared with in the non-pregnant reference group, nor were differences in drug concentrations identified by gestational age. Although there was a 38% reduction in exposure to the renally eliminated sofosbuvir metabolite GS-331007 in pregnant women compared with non-pregnant women, probably due to increased glomerular filtration rate during pregnancy,^20^ this result is not considered to be clinically significant because GS-331007 is an inactive metabolite and does not contribute to efficacy. Compared with the reference group, we observed numerically higher unbound sofosbuvir, which is not expected to be clinically relevant, and unexpectedly lower unbound ledipasvir in pregnant women. Importantly, there was a 100% cure rate among the participants in the study, with a greater than 4 log decline in viral load within 10–21 days after treatment initiation. The 100% cure rate was achieved, despite one participant missing 15 doses of ledipasvir–sofosbuvir around weeks 9 and 10 of treatment because of an opioid use relapse. Among nine maternal participants and nine infant participants, there were no clinically significant safety concerns identified with regards to maternal health, pregnancy outcomes, and infant health, including neurodevelopment, that could be contributed to ledipasvir–sofosbuvir.

The timing of perinatal HCV transmission is uncertain, but it is thought that at least a third of HCV transmissions occur antenatally before the onset of labour.^21^ Thus, initiation of treatment in the second trimester could plausibly reduce the risk of antenatal transmission and could prevent all intrapartum HCV transmission. Although it is prudent to limit medication use during pregnancy, the use of ledipasvir–sofosbuvir during pregnancy is one situation in which maternal benefits of the medication use are likely to outweigh the unknown risk of fetal exposure. Ledipasvir–sofosbuvir was well tolerated and there were no substantial safety concerns identified in this study. These findings of safety are similar to those recently reported by El-Sayed and colleagues.^22^ In El-Sayed and colleagues’ study, of seven women with first trimester daclatasvir–sofosbuvir exposure, all had full-term deliveries of infants without congenital anomalies. Six of the women discontinued daclatasvir–sofosbuvir at week 4 and one discontinued at week 8. Only the woman who discontinued at week 8 had a sustained virological response. Notably, one perinatal HCV transmission occurred in a woman who did not have a sustained virological response.^22^

Antiviral drugs are used in pregnancy for two primary reasons: to prevent perinatal infection and to treat maternal infection. For women with chronic infection in whom cure is impossible or challenging, such as women with HIV or hepatitis B virus infections, the use of antiviral medications in pregnancy is focused on prevention of perinatal infection. However, cure is highly likely with HCV direct-acting antiviral drugs. Therefore, in this study, we chose to evaluate the a complete therapeutic course because of the substantial maternal health benefits provided through prevention of cirrhosis and hepatocellular carcinoma in the mother, while simultaneously preventing perinatal HCV transmission. Although the risk of perinatal HCV transmission is low (4–8%), several studies describe that perinatal HCV transmission is often not tested for, resulting in missing cases of paediatric HCV infection.^23,24^ Even though direct-acting antiviral drug regimens are now being developed for children as young as 3 years, many children will continue to go undiagnosed. It remains unclear what effect active HCV infection might have on development.^25,26^ Antenatal HCV treatment could eradicate maternal HCV infection and prevent the sequalae of HCV infection not only in the current pregnancy, but also in future pregnancies.

This phase 1 evaluation of the pharmacokinetics and safety of ledipasvir–sofosbuvir for 12 weeks during pregnancy substantially expands our knowledge of HCV treatment during pregnancy and suggests that the pharmacokinetics of ledipasvir–sofosbuvir are similar in pregnant and non-pregnant women. However, there are limitations and challenges to this study that warrant attention. The small sample size precluded our ability to detect infrequent maternal or neonatal adverse events associated with ledipasvir and sofosbuvir exposure or a small reduction in the efficacy of ledipasvir–sofosbuvir when administered during pregnancy. Future studies should carefully choose an appropriate control group because there are coexisting factors, including poverty, substance use, and opioid exposure, that could have an effect on safety outcomes.^27^

The small sample size was directly related to the strict eligibility criteria for this study. First, the most substantial barrier to study recruitment and enrolment was the inability to confirm that women had chronic HCV infection (with a positive HCV test at least 6 months before enrolment) because many women were newly diagnosed during prenatal care screening. Second, there was an unexpectedly high prevalence of HCV genotypes 2 and 3, which accounted for ten (34%) of 29 women who were excluded after screening. Finally, the majority of women with HCV have a history of injection drug use and have psychological and social comorbidities, including housing instability, lack of childcare and transportation, and ongoing illicit drug use that precluded their participation in this time-intensive study. These strict eligibility criteria might limit the generalisability of our findings to the entire population of pregnant women with HCV infection.

Although this study had a small sample size, the implications of our findings for progressing towards the goal of HCV eradication are substantial. The results of this study suggest that no dose adjustment is needed for administration of ledipasvir–sofosbuvir during pregnancy to achieve therapeutic concentrations; therefore, larger studies evaluating the safety and efficacy of ledipasvir–sofosbuvir in pregnancy should move forward. Because ledipasvir–sofosbuvir is only approved for HCV genotypes 1, 4, 5, and 6, the pharmacokinetic evaluation of a pangenotypic regimen should concurrently be considered. Pregnancy is an ideal time for both HCV screening^28^ and treatment because of increased engagement in the health-care system and enhanced maternal investment in neonatal health outcomes. Both the US Centers for Disease Control and Prevention (on April 10, 2020)^29^ and the US Preventive Services Task Force (on March 2, 2020)^30^ recommended universal HCV screening in pregnancy, so that all pregnant women can know their HCV status. Women who are found to have HCV can be linked to HCV treatment and their infants can be appropriately screened. Our study shows that HCV screening and treatment during pregnancy are possible and provides a fundamental step towards the development of a safe and effective clinical pathway for treating HCV during pregnancy.

## Supplementary Material

Supplementary figure and protocol

## Figures and Tables

**Table 1:** Characteristics of the HCV-infected pregnant women at baseline

	HCV-infected pregnant women (n=9)
Age, years	31 (25–38)
Number of previous pregnancies	2 (0–3)
Previous livebirths	4 (44%)
Previous caesarean delivery	2 (22%)
White race	9 (100%)
Married	1 (11%)
Insurance	
Public	8 (89%)
Military	1 (11%)
Education	
Bachelor’s degree	1 (11%)
Some college	5 (56%)
High school	1 (11%)
Less than high school	2 (22%)
Primary source of income	
Work	3 (33%)
Public assistance	4 (44%)
Supported by family or partner	2 (22%)
Living situation	
Rents home	6 (67%)
Lives in home owned by family or partner	3 (33%)
Lives with partner	6 (67%)
Partner with diagnosis of HCV	2 (22%)
Current tobacco use	7 (78%)
Used recreational drugs ever	9 (100%)
Marijuana	8 (89%)
Cocaine	6 (67%)
Methamphetamines	5 (56%)
Opioids	8 (89%)
Used recreational drugs in the past year	7 (78%)
Marijuana	4 (44%)
Cocaine	5 (56%)
Methamphetamines	1 (11%)
Opioids	5 (56%)
Injection drug use in the past year	5 (56%)
Currently on opioid maintenance therapy	4 (44%)
Methadone	2 (22%)
Buprenorphine	2 (22%)
Traded sex for drugs	6 (67%)
Sexual partners	
Men only	7 (78%)
Both men and women	2 (22%)
Number of lifetime partners	
≥15	5 (56%)
7–14	3 (33%)
3–6	1 (11%)
Male partner condom use in the past year	
Never	7 (78%)
Most of the time	2 (22%)
History of anal sex	5 (56%)
Condom use with anal sex	1 (11%)
History of incarceration	8 (89%)
Incarceration in last year	2 (22%)
Has tattoos	8 (89%)
Received at least one unprofessional tattoo	5 (56%)
Route of HCV acquisition	
Intravenous drug use	8 (89%)
Perinatal	1 (11%)
Location of HCV diagnosis	
Primary care provider	2 (22%)
Inpatient hospitalisation	1 (11%)
Incarceration	1 (11%)
Drug rehabilitation programme	4 (44%)
Blood donation	1 (11%)
Linked to care for HCV diagnosis	6 (67%)
Offered HCV treatment	3 (33%)

Data are median (range) or n (%). HCV=hepatitis C virus.

**Table 2:** Sofosbuvir, GS-331007, and ledipasvir exposure in HCV-infected pregnant women

	25–26 weeks of gestation (n=8)*	29–30 weeks of gestation (n=8)*	33–34 weeks of gestation (n=8)*
**Sofosbuvir**			
AUC_tau_, h × ng/mL	1700 (20·2%)	1900 (21·6%)	1800 (20·6%)
C_max_, ng/mL	1160 (33·1%)	1270 (41·1%)	1150 (40·8%)
**GS-331007**			
AUC_tau_, h × ng/mL	9450 (14·4%)	8460 (17·0%)	8660 (11·6%)
C_max_, ng/mL	764 (20·5%)	638 (17·4%)	682 (21·7%)
**Ledipasvir**			
AUC_tau_, h × ng/mL	9500 (31·1%)	9740 (47·8%)	10 200 (30·9%)
C_max_, ng/mL	531 (33·6%)	528 (45·3%)	564 (33·7%)
C_tau_, ng/mL	346 (33·0%)	297 (59·3%)†	333 (37·0%)

Data are geometric mean (coefficient of variation). All data are presented to three significant figures. AUC_tau_=rea under the concentration–time curve of the dosing interval. C_max_=maximum measured concentration. C_tau_=concentration at the end of the dosing interval.

* One participant was excluded from the analysis because of suspected dosing errors before pharmacokinetic visits.

† n=7 (ledipasvir C_tau_ for one participant was excluded because of a suspected dosing error before this pharmacokinetic visit).

**Table 3:** Sofosbuvir, GS-331007, and ledipasvir exposure in HCV-infected pregnant women compared with non-pregnant women

	HCV-infected pregnant women (n=8)*†	HCV-infected non-pregnant women (n=43)‡	Geometric mean ratio (90% CI)
**Sofosbuvir**			
AUC_tau_, h × ng/mL	1801 (15·2%)	1977 (49·9%)	91·1% (78·0–106·3)
C_max_, ng/mL	1193 (30·1%)	1388 (47·8%)	85·9% (66·1–111·8)
**GS-331007**			
AUC_tau_, h × ng/mL	8850 (11·9%)	13 400 (29·6%)	62·0% (56·3–68·4)
C_max_, ng/mL	693 (14·4%)	1100 (25·9%)	63·1% (56·9–69·9)
**Ledipasvir**			
AUC_tau_, h × ng/mL	9800 (34·5%)	11 000 (46·0%)	89·3% (68·7–116·1)
C_max_, ng/mL	541 (36·5%)	593 (41·9%)	91·1% (70·5–117·8)
C_tau_, ng/mL	315 (37·3%)	377 (54·9%)	83·5% (63·0–110·7)

Data are geometric mean (coefficient of variation) unless otherwise stated. AUC_tau_=area under the concentration–time curve of the dosing interval. C_max_=maximum measured concentration. C_tau_=concentration at the end of the dosing interval.

* Geometric mean of individual participant pharmacokinetic parameters from all three pharmacokinetic visits were used to represent the exposure during pregnancy.

† One participant was excluded from the analysis for suspected dosing errors before pharmacokinetic visits.

‡ Non-pregnant women from ledipasvir–sofosbuvir phase 2 and 3 studies with intensive pharmacokinetic assessments.

**Table 4:** Maternal adverse events and pregnancy outcomes

	HCV-infected pregnant women (n=9)
Maternal adverse events related to ledipasvir–sofosbuvir*	5 (56%)
Maternal adverse events >grade 2 related to ledipasvir–sofosbuvir	0
Discontinuation of ledipasvir–sofosbuvir because of adverse events	0
Gestational age at delivery, weeks + days	39 + 2 (36 + 6 to 41 + 0)
Vaginal delivery	5 (56%)
Scheduled caesarean section	3 (33%)
Emergent caesarean section†	1 (11%)
Apgar score at 1 min	8 (6 to 9)
Apgar score at 5 min	9 (8 to 9)
Male infants	7 (78%)
Infant birthweight, kg	3·29 (2·60 to 4·16)
Detectable HCV RNA in cord blood	0
Length of stay in hospital, days	3 (2 to 12)
Admission to the neonatal intensive care unit‡	3 (33%)

Data are n (%) or median (range). HCV=hepatitis C virus.

* Four were grade 1 (three nausea or vomiting and one diarrhoea) and one was grade 2 (fatigue).

† Due to umbilical cord prolapse.

‡ Reasons for neonatal intensive care admission: one shoulder dystocia and two neonatal opioid withdrawal syndromes.

## Research in context

### Evidence before this study

Hepatitis C virus (HCV) infection is one of the most consequential impacts of the opioid epidemic among injection drug users. As a result, the prevalence of HCV has escalated among a growing population of pregnant women with opioid use disorder. Despite perinatal HCV transmission rates ranging from 4% to 8%, there are no interventions proven to reduce the risk of perinatal HCV transmission. The past decade has brought a revolution in treatment options for HCV, with several US Food and Drug Administration approved direct-acting antiviral drug combinations, which result in a 96–99% cure rate after only 8–12 weeks of therapy, are orally administered, and well tolerated. These new treatments offer an opportunity to treat maternal HCV infection while the woman is engaged in prenatal care and act to prevent perinatal transmission of HCV. However, as of April 13, 2020, to the best of our knowledge, there are no published studies on the safety or efficacy of direct-acting antiviral drugs for the treatment of HCV during pregnancy. We searched the MEDLINE and PubMed databases from Jan 1, 1996, to April 13, 2020, for clinical trials published in English, using the search terms “hepatitis C virus” and “pregnancy”.

### Added value of this study

To the best of our knowledge, this is the first clinical trial of HCV treatment during pregnancy with the direct-acting antiviral combination ledipasvir–sofosbuvir. We enrolled nine pregnant women with HCV who completed a 12-week course of ledipasvir–sofosbuvir starting at 23 weeks of gestation. At 12 weeks post-treatment, all the participants were cured of HCV without any clinically significant pharmacokinetic changes with ledipasvir or sofosbuvir administered during pregnancy. All infants were HCV negative and no maternal or neonatal safety concerns were identified.

### Implications of all the available evidence

These data suggest that administration of ledipasvir–sofosbuvir starting at 23 weeks of gestation is safe and effective for HCV cure. However, a larger effectiveness study is warranted before this strategy can be recommended and incorporated into routine clinical practice. Further, in this study, 34% of pregnant women screened were ineligible for participation because they had genotype 2 or 3 infection; therefore, evaluation of a pangenotypic regimen should be considered.
