# Supplementary material for: Ledipasvir plus sofosbuvir in pregnant women with hepatitis C virus infection: a phase 1 pharmacokinetic study
Source: Lancet Microbe. Author manuscript; Available in PMC 2020 Sep 15. (PMC7491553; doi:10.1016/S2666-5247(20)30062-8)
Supplement: Supplementary figure and protocol [file NIHMS1619162-supplement-Supplementary_figure_and_protocol.pdf]

Supplementary Figure 1: Individual Participant Time-Concentration Curves between 25-26 weeks', 29-30 weeks' and 33-34 weeks' for a) Sofosbuvir, b) GS-331007, and c) Ledipasvir (ng/mL)

a)

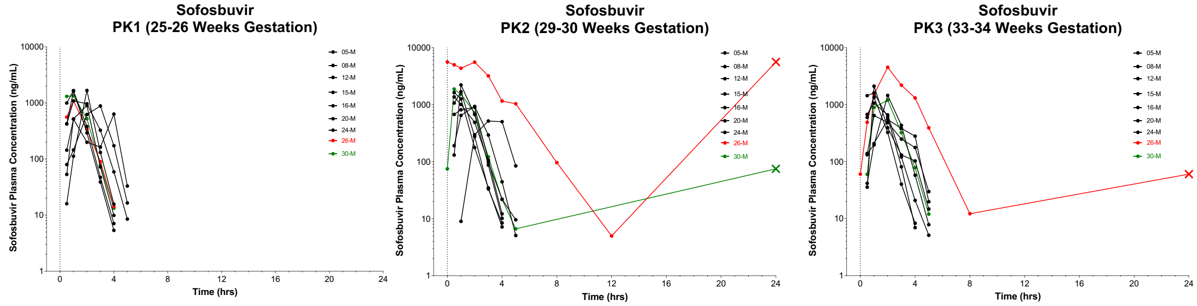

b)

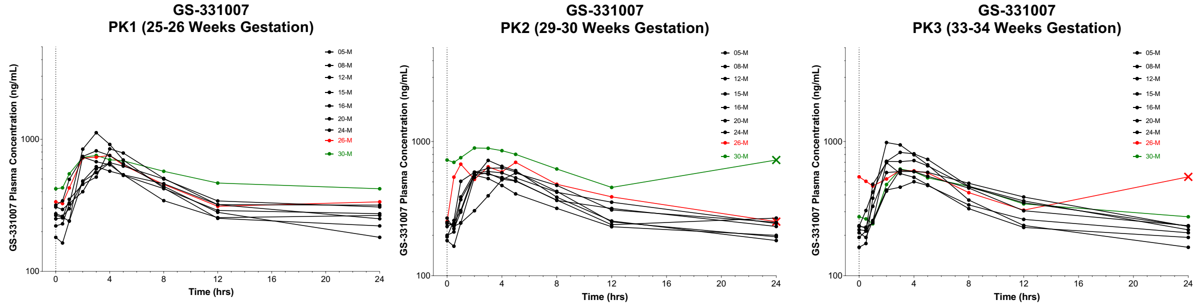

c)

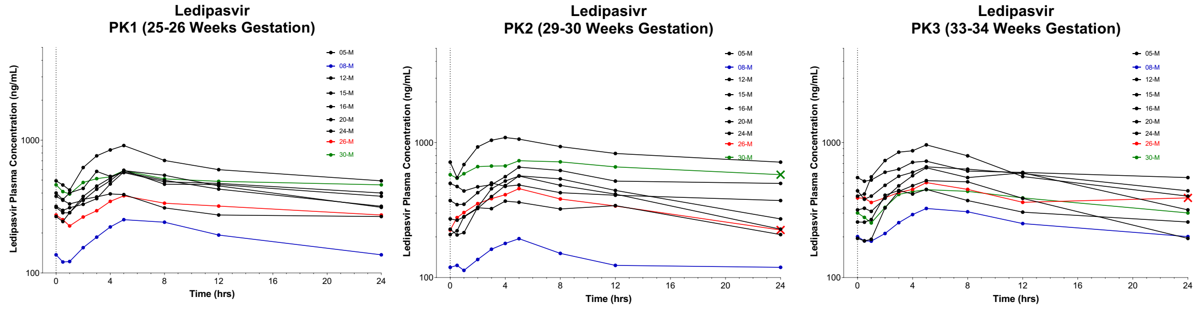

Phase 1 Pharmacokinetic Trial of Ledipasvir/Sofosbuvir Fixed Dose Combination in Pregnant Women with Chronic Hepatitis C Virus Infection

Protocol version 7.0

Funding Agencies:
Gilead Sciences CO-US-337-2117

National Institutes of Health: K12HD043441
Building Interdisciplinary Research Careers in Women's Health in Pittsburgh
(Salary Support for Dr. Chappell)

National Institutes of Health: 1R21HD089457-01
Eunice Kennedy Shriver National Institute of Child Health & Human Development

IND #: 129502

Phase 1 Pharmacokinetic Trial of Ledipasvir/Sofosbuvir Fixed Dose Combination in Pregnant Women with Chronic Hepatitis C Virus Infection

TABLE OF CONTENTS

LIST OF ABBREVIATIONS AND ACRONYMS..5
PROTOCOL TEAM ROSTER 6
Phase 1 Pharmacokinetic Trial of Sofosbuvir and Ledipasvir Fixed Dose Combination in Pregnant Women with Chronic Hepatitis C Virus Infection PROTOCOL SUMMARY..8
1 KEY ROLES..11
1.1 Protocol Identification..11
1.2 Funders, Sponsor and Monitor Identification...11
1.3 Clinical Laboratories 12
2 INTRODUCTION 12
2.1 Chronic Hepatitis C Infection in Pregnancy...13
2.2 Rationale for Treatment During Pregnancy..13
2.3 LDV/SOF Fixed Dose Combination 14
2.4 Clinical Studies 15
2.5 Rationale for Study Design 17
3 OBJECTIVES..18
3.1 Primary Objectives..18
3.2 Secondary Objectives..18
4 STUDY DESIGN 18
4.1 Identification of Study Design..18
4.2 Summary of Major Endpoints..18
4.3 Description of Study Population..19
4.4 Time to Complete Accrual..19
4.5 Expected Duration of Participation..19
5 STUDY POPULATION..20
5.1 Selection of the Study Population 20
5.2 Inclusion Criteria 21
5.3 Exclusion Criteria..21
6 STUDY MEDICATION 23
6.1 Regimen..23
6.2 Administration 23
6.3 Supply and Accountability..23
6.4 Concomitant Medications..24
7 STUDY PROCEDURES..24
7.1 Pre-screening..24
7.2 Visit 1- Screening..25
7.3 Visit 2- Enrollment..26
7.4 Follow-up Visits..26
7.5 Follow-up Procedures for Participants Permanently Discontinue Study Medication..32

7.6 Pharmacokinetics....32
7.7 Adherence Counseling and Assessment....33
7.8 Clinical Evaluations and Procedures....34
7.9 Laboratory Evaluations....35
7.10 Specimen Collection and Processing....35
7.11 Biohazard Containment....36
8 ASSESSMENT OF SAFETY....36
8.1 Safety Monitoring....36
8.2 Clinical Data and Safety Review....36
8.3 Adverse Events Definitions and Reporting Requirements....37
8.4 Expedited Adverse Event Reporting Requirements....38
8.5 Regulatory Requirements....38
8.6 Social Harms Reporting....38
9 CLINICAL MANAGEMENT....39
9.1 Grading System....39
9.2 Dose Modification Instructions....39
9.3 General Criteria for Temporary Hold and Permanent Discontinuation of Study Medication....39
9.4 Temporary Medication Hold/Permanent Discontinuation in Response to Adverse Events....40
9.5 HIV-1 Infection....40
9.6 Criteria for Early Termination of Study Participation....40
10 STATISTICAL CONSIDERATIONS....41
10.1 Overview and Summary of Design....35
10.2 Study Endpoints....36
10.3 Sample Size and Power Calculations....36
10.4 Participant Accrual, Follow-up and Retention....37
10.5 Data Analysis....37
11 DATA HANDLING AND RECORDKEEPING....44
11.1 Data Management Responsibilities....44
11.2 Source Documents and Access to Source Data/Documents....44
11.3 Quality Control and Quality Assurance....44
12 CLINICAL SITE MONITORING....44
13 HUMAN SUBJECTS PROTECTIONS....45
13.1 Institutional Review Boards....45
13.2 Study Coordination....46
13.3 Risk Benefit Statement....46
13.4 Informed Consent Process....48
13.5 Participant Confidentiality....49
13.6 Special Populations....49
13.7 Compensation....50
13.8 Communicable Disease Reporting....50
13.9 Access to HIV-related Care....50
13.10 Study Discontinuation....50
14 PUBLICATION POLICY....51
15 APPENDICES....51

APPENDIX I: SCHEDULE OF STUDY VISITS AND EVALUATIONS (Mother) 52
APPENDIX II: SCHEDULE OF STUDY VISITS AND EVALUATIONS (Infant) 46

Table of Figures

Table 1: Study Visit Schedule 10
Table 2: Clinical Studies of LDV/SOF........ 16
Table 3: Screening Visit (V1)........ 25
Table 4: Enrollment Visit (V2)........ 26
Table 5: Visits 3-5 (PK1, PK2, PK3) 27
Table 6: Delivery Visit........ 30
Table 7: Post Treatment Visit (V6) 28
Table 8:Infant Follow-up Visits (iV1, iV2, iV3) 28
Table 9:SOF and LDV PK Collection........ 29

Phase 1 Pharmacokinetic Trial of Sofosbuvir and Ledipasvir Fixed Dose Combination in Pregnant Women with Chronic Hepatitis C Virus Infection

LIST OF ABBREVIATIONS AND ACRONYMS

ALT	Alanine tranaminase
AST	Aspartate transaminase
AUC	Area under the curve
DAIDS	Division of AIDS
DHHS	Department of Health and Human Services
DNA	deoxyribonucleic acid
DSMB	Data and Safety Monitoring Board
EAE	Expedited adverse event
FDA	Food and Drug Administration
GCLP	Good Clinical Laboratory Practices
GCP	Good Clinical Practices
HBsAg	Hepatitis B surface antigen
HBV	Hepatitis B Virus
HCV	Hepatitis C Virus
HIV	Human immunodeficiency virus
INR	International normalized ratio
IoR	Investigator of Record
IRB	Institutional Review Board
ISR	Interim Study Review
LDV	Ledipasvir
LDV/SOF FDC	Ledipasvir and sofosbuvir fixed-dose combination
mL	Milliliter
NIAID	National Institute of Allergy and Infectious Disease
NIH	National Institutes of Health
OHRP	Office of Human Research Protections
PoR	Pharmacist of Record
PTID	Participant identification number
RNA	Ribonucleic acid
SAE	Serious adverse event
SOF	Sofosbuvir
SOP	Standard operating procedure(s)
SSP	Study-specific Procedures
UPMC	University of Pittsburgh Medical Center

Phase 1 Pharmacokinetic Trial of Sofosbuvir and Ledipasvir Fixed Dose Combination in Pregnant Women with Chronic Hepatitis C Virus Infection

PROTOCOL TEAM ROSTER

University of Pittsburgh Study Team

Catherine Chappell, MD, MSc
Magee-Womens Hospital of UPMC
300 Halket Street
Pittsburgh, PA 15213 USA
Phone: 412-641-1809
Fax: 412-641-1133
Email: chappellca@upmc.edu

Sharon Hillier, PhD
Magee-Womens Research Institute
204 Craft Avenue
Pittsburgh, PA 15213 USA
Phone: 412-641-8933
Fax: 412-641-6170
Email: shillier@mail.magee.edu

John Mellors, MD, MPH
Division of Infectious Disease
University of Pittsburgh School of Medicine
Scaife Hall, Suite 818
3550 Terrace Street
Pittsburgh, PA 15261
Phone: 412-624-8512
Fax: 412-383-7982
Email: mellors@dom.pitt.edu

Richard Beigi, MD, MSc
Magee-Womens Hospital of UPMC
300 Halket Street
Pittsburgh, PA 15213 USA
Phone: 412-641-3313
Fax: 412-641-1133
Email: rbeigi@mail.magee.edu

Elizabeth Krans, MD, MSc
Magee-Womens Hospital of UPMC
300 Halket Street
Pittsburgh, PA 15213 USA
Phone: 412-641-4222
Fax: 412-641-1133
Email: kransee@mail.magee.edu

Steve Caritis, MD
Magee-Womens Hospital of UPMC
300 Halket Street
Pittsburgh, PA 15213 USA
Phone: 412-641-4874
Fax: 412-641-1133
Email: scaritis@mail.magee.edu

Gilead Sciences Study Team

John McHutchinson, MD
303 Velocity Way, 9th Floor
Foster City, CA 94404
Phone: 650-522-5302
Email: john.mchutchison@gilead.com

Diana Brainard, MD
303 Velocity Way, 9th Floor
Foster City, CA 94404
Phone: 650-522-4761
Email: diana.brainard@gilead.com

Mani Subramanian, MD, PhD
303 Velocity Way, 9th Floor
Foster City, CA 94404
Phone: 650-522-6084
Email: mani.subramanian@gilead.com

Anita Mathias, PhD
333 Lakeside Drive
Foster City, CA 94404
Phone: 650-522-6375
Email: anita.mathias@gilead.com

Brian Kirby, PhD
333 Lakeside Drive
Foster City, CA 94404
Phone: 650-522-2806
Email: brian.kirby@gilead.com

Phase 1 Pharmacokinetic Trial of Ledipasvir/Sofosbuvir Fixed Dose Combination in Pregnant Women with Chronic Hepatitis C Virus Infection

PROTOCOL SUMMARY

**Short Title:**	Treatment of Chronic Hepatitis C during Pregnancy with Ledpasvir/Sofosbuvir
**Clinical Phase:**	Phase 1
**IND Sponsor:**	Catherine Chappell, MD, MS
**Protocol Chair:**	Catherine Chappell, MD, MS
**Sample Size:**	15 women and their infants (participants who do not complete treatment or report less than 85% adherence will be replaced)
**Study Population:**	Pregnant women diagnosed with genotype 1, 4, 5, or 6 chronic hepatitis C infection that are between the ages of 18 and 39 and are 14 + 0 and 22 + 6 weeks of gestation at Screening
**Study Site:**	Magee-Womens Hospital of UPMC, Pittsburgh, PA
**Study Design:**	Single-site, single-arm pilot study
**Study Duration:**	Approximately 28 weeks per maternal participant and one year of infant follow up.
**Study Medication:**	Fixed-dose combination tablet of Ledipasvir 90 mg and Sofosbuvir 400 mg (LDV/SOF FDC)
**Study Regimen:**	Participants will take LDV/SOF FDC one tablet once daily starting between 23 + 0 and 24 + 6 weeks of gestation for 12 weeks total.
**PK Sampling:**	Systemic exposure both LDV and SOF (SOF and inactive metabolite GS-331007) will be assessed at 3 gestational age time points:<br>1) Late second trimester between 25 + 0 and 26 + 6 weeks' gestation (after at least 10 days of therapy);<br>2) Early third trimester between 29 + 0 and 30 + 6 weeks' gestation;<br>3) Late third trimester between 33 + 0 and 34 + 6 weeks' gestation

Primary Objective:

- To compare the pharmacokinetic (PK) parameters of LDV/SOF FDC at different gestational ages during pregnancy to available data from nonpregnant women.

Primary Endpoint:

- Systemic plasma exposures of both LDV and SOF (SOF and GS-331007) will be assessed in:
  - Late second trimester (25 + 0 to 26 + 6 weeks)
  - Early third trimester (29 + 0 to 30 + 6 weeks)
  - Late third trimester (33 + 0 to 34 + 6 weeks).

Secondary Objectives:

- To determine the rate of sustained viral response (SVR12), defined by undetectable hepatitis C virus twelve weeks after completion of LDV/SOF FDC treatment for 12 weeks during pregnancy.

- To evaluate the safety for the mother and for the neonate of hepatitis C treatment with LDV/SOF during pregnancy.

Secondary Endpoints:

- **Sustained Viral Response (SVR12)**
  - HCV RNA viral load will be assessed at 12 weeks after completion of LDV/SOF treatment. An undetectable viral load will be considered a SVR12
- **Maternal Safety**
  - Safety laboratory assessments of blood counts and liver function will be measured at the PK1 and PK2 visits (approximately 2-4 weeks and 6-8 weeks of treatment)
  - Pregnancy and delivery outcomes collected prospectively

- **Neonate Safety**
  - Major malformations, defined as structural abnormalities with medical, surgical or cosmetic importance
  - Weight, length, and head circumference at birth, 8 weeks, six months and 12 months
  - HCV RNA viral load will be assessed at birth (as available), one to three months, at six months and then again at 12 months only if negative viral loads are not documented at one to three and six months
  - Developmental exams will be performed at six months and 12 months

Table 1: Study Visit Schedule

	Maternal Study Visit	Gestational Age (Weeks + Days)
V1	Screening	14+0 to 22+6
V2*	Enrollment	23+0 to 24+6
V3**	PK1	25+0 to 26+6†
V4	PK2	29+0 to 30+6†
V5	PK3	33+0 to 34+6†
V6***	End of Treatment	
Del****	Delivery	
V7/iV1	Post treatment	12 weeks ± 4 days after completion of therapy†
	Infant Study Visit	Infant Age
Del****	Delivery	Newborn
V7/iV1	Infant Visit 1	8 weeks ± 6 weeks†
iV2	Infant Visit 2	6 months ± 2 weeks†
iV3	Infant Visit 3	12 months ± 2 weeks†

†These are target windows. If the participant cannot be seen within these windows then it will not be considered a protocol deviation.
*Start LDV/SOF FDC and continue for 12 weeks
**Must be at least 10 days after Enrollment and initiation of LDV/SOF FDC
***Must be within 7 days of LDV/SOF FDC course completion
****The Delivery Visit will occur during admission for participants delivering at Magee-Womens Hospital. Participants who deliver at an outside location will be contacted by phone as close to delivery as possible to complete the applicable Delivery Visit study procedures (i.e. questionnaire, update locator information, update medical history). All participants, regardless of delivery location will undergo a chart review to obtain delivery outcomes/variables.

1 KEY ROLES

1.1 Protocol Identification

Protocol Title:	**Phase 1 Pharmacokinetic Trial of Ledipasvir/Sofosbuvir Fixed Dose Combination in Pregnant Women with Chronic Hepatitis C Virus Infection**
Short Title:	Treatment of Chronic Hepatitis C during Pregnancy with Ledpasvir/Sofosbuvir
Date:	October 17, 2017

1.2 Funders, Sponsor and Monitor Identification

Funding Agencies:

NIH Building Interdisciplinary Research Careers in Women's Health in Pittsburgh

Gilead Sciences

National Institutes of Health Eunice Kennedy Shriver National Institute of Child Health & Human Development

IND Sponsor: Catherine Chappell, MD MSc

Site Monitoring: Catherine Chappell, MD MSc

Pharmaceutical Collaborator: Gilead Sciences

1.3 Clinical Laboratories

Laboratory Center:	UPMC Presbyterian Shadyside CPPUH<br>UPMC Clinical Laboratory Building<br>3477 Euler Way<br>Pittsburgh, PA 15213 USA
Pharmacology (Primary PK):	Sample Team<br>Delaware Technology Park<br>Three Innovation Way, Suite 240<br>Newark, DE 19711
Pharmacology (Protein Binding):	QPS, LLC<br>Pencader Corporate Center<br>110 Executive Drive, Suite 7<br>Newark, DE 19702
HCV Resistance Testing:	Quest Diagnostics Incorporated<br>1901 Sulphur Spring Rd.<br>Baltimore MD 21227

2 INTRODUCTION

2.1 Chronic Hepatitis C Infection in Pregnancy

Hepatitis C is highly prevalent and causes significant morbidity and mortality. More than 130 million people have chronic HCV infection[1], including 3.2 million infected in the United States[2] with an estimated incidence during pregnancy of 1-2.4%[3]. Chronic hepatitis C is the most common cause of chronic liver disease and cirrhosis, and the most common indication for liver transplantation in the United States[4]. It was previously thought that HCV had limited effects on pregnancy. However, recent reports have shown an association with intrahepatic cholestasis of pregnancy, gestational diabetes, and preterm delivery in seropositive gravidas[5-8]. HCV viral load increases in the second and third trimester of pregnancy, a factor which likely enhances the risk of perinatal transmission[9,10]. In addition to the risk of perinatal infection, the adverse fetal outcomes reported include an increased incidence of congenital anomalies, low birth weight, newborns small for gestational age, need for assisted ventilation, and requirement of neonatal intensive care[5]. Most recent data suggest that HCV seropositivity during pregnancy is associated with an adverse neurologic outcome, cephalohematoma, fetal distress, feeding difficulties, intraventricular hemorrhage, and neonatal seizure[11].

Cure of HCV provides significant health benefits. The goal of HCV treatment is to reduce the burden of HCV so that it is no longer detectable in any tissue, including the serum, liver tissue and mononuclear cells[12-14]. Sustained virologic response (SVR12) is defined as the absence of detectable HCV RNA in the serum at least 12

weeks after ending therapy. SVR12 has been shown to be a durable predictor of HCV cure followed up for five years or more[15,16]. Patients cured of HCV experience significant health benefits including decreased liver inflammation, slowed and reversed rates of progression of liver fibrosis and cirrhosis[17]. Additionally, SVR12 is associated with a 70% reduction in risk of hepatocellular carcinoma and a 90% reduction in the risk of liver-related mortality and liver transplantation[18,19]. Not only does HCV cure reduce liver disease, it also reduces morbidity and mortality from extrahepatic manifestation of HCV infection, including cryoglobulinemic vasculitis[20], non-Hodgkin lymphoma[21] and other lymphoproliferative disorders[22]. The most recent HCV treatment guidelines recommend treatment in all HCV-infected persons, except those with limited life expectancy due to non-liver related comorbid conditions[23].

Direct-acting antiviral agents are highly effective in the treatment of chronic HCV infection. Previous treatments for chronic HCV infection included pegylated-interferon and ribavirin produced SVR12 of only 40-50% for patients infected with genotype 1 (the most common genotype in the United States) despite prolonged treatment durations of 48 weeks[24-26]. Pegylated-interferon does not act directly on HCV but instead activates immune mechanisms of viral clearance, which in turn leads to an unfavorable side effect profile and are contraindicated in women of reproductive age that are pregnant or planning pregnancy. HCV-encoded proteins required for viral replication have, therefore, become the major focus of new therapies in the treatment of chronic HCV and have heralded the era of the direct-acting antivirals (DAAs). The HCV RNA genome encodes a single polyprotein of ~3000 amino acids that require cleavage by both host and viral proteases to form three structural proteins and seven non-structural proteins.

The recent October 2014 approval of the fixed dose combination of the NS5B polymerase inhibitor sofosbuvir (SOF) and the NS5A inhibitor ledipasvir (LDV) for the treatment of treatment-naive and -experienced HCV genotype 1a/1b (HCV-1) has marked a new era of IFN and ribavirin free treatment for chronic hepatitis C. LDV/SOF combination is approved for 12 weeks in treatment-naive patients with and without cirrhosis. For treatment-experienced patients, it is approved for 12 weeks in patients without cirrhosis but for 24 weeks in patients with cirrhosis. A shorter 8-week course of treatment can be considered for treatment-naive patients who have pretreatment HCV RNA of < 6 million IU/ml and do not have cirrhosis[23,27]. The fixed-dose combination (FDC) of LDV (90 mg) and SOF (400 mg) has a SVR12 of approximately 96% when given as a once-a-day pill for 3 months to both treatment-naive and -experienced HCV-1 patients with the exception of prior null responders with cirrhosis[27-29]. Since FDA approval, LDV/SOF is considered first line initial treatment for HCV genotype 1a/1b, and also for genotypes 4, 5, and 6, with similar SVR12 to genotype 1a/1b: 95% (N=43)[30,31], 95% (N=41)[32], and 96% (N=25)[33], respectively[23].

2.2 Rationale

Pregnant women would be uniquely motivated to be adherent to the treatment regimen and to not become re-infected postpartum. The HCV treatment guidelines recommend that priority consideration be given to treatment of Reproductive-aged women who desire pregnancy[23]. A recent meta-analysis of the risk of perinatal HCV infection to children of HCV antibody-positive and RNA-positive women was 5.8% (95% confidence interval [CI], 4.2%-7.8%) for children of HIV-negative women and 10.8% (95% CI, 7.6%-15.2%) for children of women co-infected with human immunodeficiency virus (HIV)[34]. Women with chronic HIV infection are more likely to adhere to antiviral treatment during the antepartum period as compared to the postpartum period. A meta-analysis of adherence to antiretroviral therapy during and after pregnancy in low-income, middle-income and high-income countries showed a statistically significant difference in adequate adherence levels 75.5% (95% CI 71.5-79.7%) in the antepartum period as compared to 53.0% (95% CI 32.8-72.7%) in the postpartum period[35]. Many HIV-infected women describe a loss of motivation to continue antiretroviral therapy postpartum and breastfeeding because it is no longer required to protect the child[36,37]. Additionally, this decrease in motivation was compounded by the overwhelming demands of everyday life[37]. We hypothesize that HCV-infected women will be similarly motivated and will be more likely to adhere to treatment during the antepartum period than outside of pregnancy or in the postpartum period.

2.3 LDV/SOF Fixed Dose Combination

2.3.1 Description

Sofosbuvir is described chemically as (S)-Isopropyl 2-((S)-(((2R,3R,4R,5R)-5- (2,4-dioxo-3,4-dihydropyrimidin-1(2H)-yl)-4-fluoro-3-hydroxy-4-methyltetrahydrofuran-2-yl)methoxy)-(phenoxy)phosphorylamino)propanoate. It has a molecular formula of $C_{22}H_{29}FN_3O_9P$ and a molecular weight of 529.45. Sofosbuvir is a white to off-white crystalline solid with a solubility of ≥2 mg/mL across the pH range of 2–7.7 at 37C and is slightly soluble in water.

Ledipasvir is described chemically as s Methyl [(2S)-1-{(6S)-6-[5-(9,9-difluoro-7-{2-[(1R,3S,4S)-2-{(2S)-2-[(methoxycarbonyl)amino]-3-methylbutanoyl}-2-azabicyclo[2.2.1]hept-3-yl]-1H-benzimidazol-6-yl}-9H-fluoren-2-yl)-1H-imidazol-2-yl]-5-azaspiro[2.4]hept-5-yl}-3-methyl-1-oxobutan-2-yl]carbamate. It has a molecular formula of $C_{49}H_{54}F_2N_8O_6$ and a molecular weight of 889.00.

Each tablet contains 90 mg ledipasvir and 400 mg sofosbuvir. The tablets include the following inactive ingredients: colloidal silicon dioxide, copovidone, croscarmellose sodium, lactose monohydrate, magnesium stearate, and microcrystalline cellulose. The tablets are film-coated with a coating material containing the following inactive ingredients: FD&C yellow #6/sunset yellow FCF aluminum lake, polyethylene glycol, polyvinyl alcohol, talc, and titanium dioxide[38].

2.3.2 Mechanism of Action

Ledipasvir inhibits the HCV NS5A protein and sofosbuvir inhibits HCV NS5B RNA-dependent RNA polymerase, which are essential for viral replication[38]. Sofosbuvir is a nucleotide prodrug that is metabolized to the pharmacologically active metabolite GS-461203, which is incorporated into HCV RNA by NS5B polymerase where it acts as a chain terminator[38].

Ledipasvir had potent antiviral activity against HCV genotypes 1a and 1b (EC50 values of 0.031 and 0.004 nmol/L in HCV replicon assays), whereas it had lower activity against HCV genotypes 4a, 4d, 5a and 6a (EC50 values of 0.39, 0.60, 0.15, and 1.1 nmol/L, respectively) and substantially lower activity against genotypes 2a, 2b, 3a and 6e (EC50 values of 21-249, 168, 264 nmol/L, respectively)[38]. Sofosbuvir demonstrated pangenotypic antiviral activity, with EC50 values against HCV genotypes 1a, 1b, 2a, 2b, 3a, 4a, 5a, and 6a, of 40, 110, 50, 15, 50, 40, 15, and 14 nmol/L, respectively in HCV replicon assays[39]. LDV/SOF has potent activity against HCV genotype 1, 4, 5, and 6, but is less effective against 2 and 3HCV genotypes[38].

2.4 Clinical Studies

2.4.1 Clinical Studies of Sofosbuvir/Ledipasvir for the Treatment of HCV

Approximately 1518 participants have been enrolled in several multicenter, Phase 3 trials to assess the efficacy of treatment with LDV/SOF in patients with genotype 1 chronic hepatitis C virus infection, ION-1, ION-2 and ION-3 (Table 2)[27-29,38]. Patients in ION-1[29] and ION-3[27] were treatment naïve and patients in ION-2[28] had experienced virological failure after treatment with peg-interferon-alpha (peg-INF) plus ribavirin (RBV) with or without a NS3/4A protease inhibitor. Patients in ION-1 and ION-2 received LDV/SOF with or without RBV for 12 or 24 weeks, and patients in ION-3 received LDV/SOF for 8 or 12 weeks. The primary endpoint for all three trials was a HCV RNA level of <25IU/mL at 12 weeks after completion of treatment (SVR12). In ION-1, SVR12 rates were >97% regardless of treatment duration (12 vs. 24 weeks) or co-administration with ribavirin. ION-3 demonstrated an SVR12 rate of 94% in treatment-naïve patients without cirrhosis who received LDV/SOF for only 8 weeks and in this study 8 weeks of therapy was noninferior to 12 weeks of therapy[27]. Post hoc analysis of ION-3 demonstrated that treatment with LDV/SOF for 8 weeks could be less effective for patients with a baseline RNA level > or equal to 6 million IU/mL[27]. Among patients with a baseline HCV RNA level of <6 and >/= 6 million, the SVR12 rates was 97 and 90% in those receiving LDV/SOF for 8 weeks, compared to 96 and 96% in those receiving LDV/SOF for 12 weeks[27].

Table 2: Clinical studies of LDV/SOF for the treatment of HCV genotype 1

Study	Regimen (Duration)	Prior Treatment	N Total	N SVR12	N Treatment Failures	Rate of SVR12 (95% CI)
ION-1	LDV/SOF (12)	No	214	211	1	99 (96-100)

	LDV/SOF + RBV (12)	No	217	217	0	97 (94-99)
	LDV/SOF (24)	No	217	212	2	98 (95-99)
	LDV/SOF + RBV (24)	No	217	215	0	99 (97-100)
ION-2	LDV/SOF (12)	PEG-INF + RBV	109	102	7	94 (87-97)
	LDV/SOF + RBV (12)	PEG-INF + RBV	111	107	4	96 (91-99)
	LDV/SOF (24)	PEG-INF + RBV	109	108	0	99 (95-100)
	LDV/SOF + RBV (24)	PEG-INF + RBV	111	110	1	99 (95-100)
ION-3	LDV/SOF (8)	No	215	202	11	94 (90-97)
	LDV/SOF + RBV (8)	No	216	201	9	93 (89-96)
	LDV/SOF (12)	No	216	206	3	95 (92-98)

Clinical studies of HCV genotype 4 treatment with LDV/SOF include two open-label studies of 43 participants. The SYNERGY trial evaluated 12 weeks of LDV/SOF in 21 patients, of whom 60% were treatment-naïve and 43% had advanced fibrosis. One participant withdrew consent, but the rest achieved SVR12 (20/21)[26]. The other study enrolled 22 treatment naïve genotype 4 patients, 21 achieved SVR12 with a 12 week course of LDV/SOF[8]. Similarly, an open-label study of 41 patient with HCV genotype 5 infection were treated with a 12 week course of LDV/SOF and 95% achieved SVR12[7]. A small, two-center, open-label study investigated efficacy of ledipasvir/sofosbuvir for 12 weeks in treatment-naïve and -experienced patients with HCV genotype 6 infection with 24 of 25 patients achieving SVR12 (96%)[18].

Safety

The safety and tolerability of LDV/SOF has been evaluated within the setting of Phase 3 clinical trials. Overall, LDV/SOF was well tolerated by patients with chronic HCV infection (N=1080) with the incidence of adverse events of 74% overall and 45% that were treatment-related[38]. The proportion of patients who permanently discontinued treatment due to adverse events was 0%, <1% and 1% when receiving LDV/SOF for 8, 12, and 24 weeks, respectively[38,40]. Treatment discontinuation was much more common with LDV/SOF when RBV was co-administered. The most common adverse events reported with LDV/SOF alone were fatigue, headache, nausea, diarrhea and insomnia; the majority of adverse events were mild to moderate. Bilirubin elevations of 1.5x the upper limit of normal were observed in 3%, <1% and 2% of patients treated with LDV/SOF for 8, 12, and 24 weeks, respectively. Transient, asymptomatic lipase elevations of greater than 3x the upper limit of normal were observed in <1%, 2%, and 3% of participants treated with LDV/SOF for 8, 12, and 24 weeks, respectively[38,40].

2.4.2 Animal Studies of Sofosbuvir and Ledipasvir During Pregnancy

There are no adequate studies of LDV/SOF in pregnant women. Currently, the LDV/SOF package insert recommends that it should be used during pregnancy only if the potential benefit justifies the potential risk to the fetus.

Animal Data

Ledipasvir: No effects on fetal development have been observed in rats and rabbits at the highest doses tested. In the rat and rabbit, AUC exposure to ledipasvir was approximately 4- and 2-fold, respectively, the exposure in humans at the recommended clinical dose[38].

Sofosbuvir: No effects on fetal development have been observed in rats and rabbits at the highest doses tested. In the rat and rabbit, AUC exposure to the predominant circulating metabolite GS-331007 increased over the course of gestation from approximately 3- to 6-fold and 7- to 17-fold the exposure in humans at the recommended clinical dose, respectively[38].

2.5 Rationale for Study Design

There is currently no experience with LDV/SOF in pregnant women, although based on the animal model data submitted to the FDA, this drug combination was given a pregnancy category B designation, meaning that there were no effects in on fetal development observed in rats and rabbits with doses higher than those given for treatment of hepatitis C in humans[38]. Before larger scale treatment studies can be undertaken, it will be necessary to assess whether the pharmacokinetics of the drugs are similar in pregnant and nonpregnant women, and whether the trajectory of viral response to treatment is similar to that observed in nonpregnant women, given the higher viral loads observed during pregnancy. This pilot study is designed to address those knowledge gaps. We propose a single-arm, open label Phase 1 study of chronic hepatitis C treatment during pregnancy of 15 HCV (genotype 1, 4, 5 or 6)-infected pregnant women with a 12-week course of LDV/SOF. We propose a 12-week course because it is known HCV RNA concentrations are higher during pregnancy, thus a longer course will decrease the risk of HCV treatment failure. We will begin treatment during the second trimester, therefore eliminating the risk of LDV/SOF exposure to the fetus during organogenesis. Additionally, this will allow adequate time for treatment course completion by delivery.

The study will be completed in 10 or 11 visits (7 maternal visits, delivery visit and 3 infant visits) which should easily align with prenatal and postpartum visits (Table 1). We plan to screen patients between 14+0 and 22+6 weeks of gestation confirmed by ultrasound by the time of their Enrollment visit who are known to be HCV antibody positive. An HCV RNA level to confirm the patient is actively infected with HCV as well as an HCV genotype will be obtained. A full laboratory evaluation of liver function including CBC, PT/INR, hepatic panel, creatinine, creatinine kinase and lipase will be obtained, to evaluate for renal failure and decompensated cirrhosis. A Hepatitis B

Virus (HBV) panel will be performed to test all patients for evidence of current or prior HBV infection before initiation of HCV treatment. Medical history and demographic information will also be collected at screening. If the following inclusion and exclusion criteria are met, then the patient will be enrolled into the study between 23+0 and 24+6 weeks' gestation and initiated on a 12 week course of LDV/SOF.

3 OBJECTIVES

3.1 Primary Objective

1. To compare the pharmacokinetic parameters of LDV/SOF FDC during pregnancy to those obtained outside of pregnancy.

3.2 Secondary Objectives

1. To determine the rate of SVR12 of hepatitis C virus genotype 1, 4, 5 or 6 with LDV/SOF FDC treatment for 12 weeks during pregnancy.

2. To evaluate the maternal and neonatal safety of hepatitis C treatment with LDV/SOF FDC during pregnancy.

4 STUDY DESIGN

4.1 Identification of Study Design

This is a single-site, open label, single-arm Phase 1 trial.

4.2 Summary of Major Endpoints

Primary Endpoint:

- **Systemic PK**
  - Systemic exposure of both LDV and SOF (SOF and inactive metabolite GS-331007) will be assessed at 3 gestational age time points:
    1) Late second trimester between 25 + 0 and 26 + 6 weeks' gestation (after at least 10 days of therapy)
    2) Early third trimester between 29 + 0 and 30 + 6 weeks' gestation
    3) Late third trimester between 33 + 0 and 34 + 6 weeks' gestation

Secondary Endpoints:

- **Sustained Viral Response (SVR12)**
  - HCV RNA viral load will be assessed at 12 weeks after LDV/SOF treatment completion. An undetectable viral load will be considered a SVR12

- **Maternal Safety**
  - Safety laboratory assessments of blood counts, creatinine, liver function, creatinine kinase and lipase will be measured at the PK1 and PK2 visit (approximately 2-4 and 6-8 weeks after initiation of LDV/SOF FDC)
  - Pregnancy and delivery outcomes collected prospectively by chart review and participant interview

- **Neonate Safety**
  - Major malformations, defined as structural abnormalities with medical, surgical or cosmetic importance
  - Weight, length, and head circumference at birth (by exam or chart review), 8 weeks, six months and 12 months
  - HCV RNA will be assessed at birth (for participants delivering at MWH), 1-3 months and 6 months, and then again at 12 months only if negative viral loads are not documented at one to three <u>and</u> six months
  - Neonatal development assessed by Bayley's development testing at six months and 12 months

4.3 Description of Study Population

The study population will include 15 pregnant women between the ages of 18-39 year old (inclusive) at Screening who are chronically infected with Hepatitis C virus, genotype 1, 4, 5 or 6, as described in Sections 5.2 and 5.3.

4.4 Time to Complete Accrual

The approximate time to complete study enrollment is expected to be 12 months.

4.5 Expected Duration of Participation

The expected duration for maternal participants is approximately 24 weeks, not including the screening window. Infants will be followed at least for a full year after birth; therefore mother-child pairs will be enrolled for approximately one year and 4 months. Maternal study data will be collected from the participant and/or her medical records through the Post Treatment Visit (V7). Maternal participants who have AEs at the Post Treatment Visit (V7) that have not resolved or stabilized will be followed beyond V7 until a clinically acceptable resolution of the AE(s) is confirmed and documented. Similarly, infant study data will be collected through the 12 month follow-up visit (iV3). Infants who have AEs at the iV3 visit that have not resolved or stabilized will be followed beyond iV3 until a clinically acceptable resolution of the AE(s) is confirmed and documented. Clinical acceptability of resolution will be determined by the Protocol Chair in consultation with the study team.

5 STUDY POPULATION

5.1 Selection of the Study Population

The inclusion and exclusion criteria in Sections 5.2 and 5.3 will be utilized to ensure the appropriate selection of study participants.

5.1.1 Recruitment

HCV-infected pregnant women will be recruited from antenatal clinics, methadone clinics and recovery programs in Pittsburgh and surrounding areas. There are over 10,000 deliveries at MWH per year and it is estimated that approximately 1% of women who deliver are infected with HCV. Recruitment will be primarily targeted from the MWH Pregnancy Recovery Center, which was established in 2014 to provide concurrent treatment for opiate dependence (buprenorphine) with prenatal care, which has an estimated retention rate of 90% for weekly opiate substitution and prenatal visits. There are approximately 120 opiate addicted pregnant women who received prenatal care at the Pregnancy Recovery Center per year. The prevalence of HCV infection in this Center is approximately 60%. Recruitment will also be targeted at the Magee-Women Hospital ambulatory clinic and the private practice clinics that deliver at Magee-Womens Hospital. These locations will capture HCV+ pregnant women who are on methadone substitution therapy or are not opiate dependent and planning to deliver at Magee-Womens Hospital. In order to engage HCV-infected women in recovery, but not yet engaged in prenatal care, we will also recruit patients from local methadone clinics and recovery programs that treat pregnant women.

Recruitment will also include women who are not receiving care at Magee-Womens Hospital through referrals from the care providers. These women must bewilling to travel to Pittsburgh to complete the study visits but may deliver at an outside hospital as information from delivery can be obtained via chart review)

Recruitment materials, including participant advertisements, will be approved by the University of Pittsburgh Institutional Review Board prior to use.

5.1.2 Retention

The importance of retention will be stressed to the participant at each visit as part of protocol adherence counseling. Once a participant is enrolled, the study staff will make every effort to retain the participants in follow-up to minimize possible bias associated with loss-to-follow-up. Any participant that discontinues the study before the PK3 visit is completed or if a participant reports missing more than 1 dose per week averaged over the duration of the study then they will be replaced by recruiting another participant into the study. We anticipate needing to replace no more than two participants.

5.2 Inclusion Criteria

Women must meet all of the following criteria to be eligible for inclusion in the study. Any exclusionary laboratory values can be repeated at a later date within the Screening window. If the repeated laboratory values meet inclusion criteria then the participant can be enrolled. If there is concern that the participant's health status has changed between the enrollment visit and the screening visit or if there is concern by the study investigators that the participant might not remain eligible, the screening laboratories can be repeated prior to enrollment.

1) Age 18 through 39 years (inclusive) at Screening

2) Able and willing to provide written informed consent to be screened for and take part in the study procedures

3) Able and willing to provide adequate locator information

4) Chronic HCV, genotype 1, 4, 5 or 6 infection, defined as HCV antibody detected at least 6 months prior to Screening and detectable HCV RNA viral load at Screening

5) Desired pregnancy at 23 + 0 to 24 + 6 weeks' gestation at Enrollment with gestational dating confirmed by ultrasound

6) Singleton gestation with no known fetal abnormalities

7) Documented negative Hepatitis B testing for current infection (negative HBsAg test) or previous infection (negative anti-HB Core) performed at the screening visit

8) Negative HIV testing at the screening visit

9) Per participant report at Screening and Enrollment, agrees not to participate in other research studies involving drugs or medical devices for the duration of study participation

5.3 Exclusion Criteria

Women who meet any of the following criteria will be excluded from the study:

1) Participant report of any of the following at Screening or Enrollment:
    a. Previous treatment for Hepatitis C virus with sofosbuvir or a NS5A inhibitor

b. Use of any medications contraindicated with concurrent use of ledipasvir or sofobuvir according to the most current HARVONI package insert
c. Plans to relocate away from the study site area in the next 1 year and 4 months
d. Current sexual partner is known to be infected with HIV or Hepatitis B virus
e. History of cirrhosis documented by previous liver biopsy or liver imaging tests

2) Reports participating in any other research study involving drugs or medical devices within 60 days or less prior to Enrollment

3) Clinically significant and habitual non-therapeutic drug abuse, not including marijuana, as determined by Protocol Chair

4) At Screening or Enrollment, as determined by the Protocol Chair, any significant uncontrolled active or chronic cardiovascular, renal, liver (such as evidence of decompensated cirrhosis by ascites, encephalopathy, or variceal hemorrhage), hematologic, neurologic, gastrointestinal, psychiatric, endocrine, respiratory, immunologic disorder or infectious disease (other than Hepatitis C)

5) Has a high risk of preterm birth defined as a history of spontaneous preterm birth at less than 34 weeks of gestation or a shortened cervical length of less than 20 millimeters

6) Has any of the following laboratory abnormalities at Screening:
   a. Aspartate aminotransferase (AST) or alanine transaminase (ALT) greater than 10 times the upper limited of normal
   b. Hemoglobin less than 9 g/dL
   c. Platelet count less than 90,000 per mm^3
   d. International normalized ratio (INR) > 1.5
   e. Creatinine greater than 1.4
   f. Medical history of cirrhosis

7) Has any other condition that, in the opinion of the IoR/designee, would preclude informed consent, make study participation unsafe, complicate interpretation of study outcome data, or otherwise interfere with achieving study objectives.

6 STUDY MEDICATION

6.1 Regimen

Each participant will take a 12 week course of LDV/SOF FDC one tablet by mouth, once daily.

6.2 Administration

Study medication will be dispensed at Enrollment, PK1, PK2, and PK3 in the quantities sufficient to have one dose per day until the next scheduled visit with 3 extra doses. Tablets will be packaged by the Investigational Pharmacist/Pharmacist of Record (PoR) in the pharmacy at Magee-Womens Hospital. Participants will be given a medication administration log to assist them with keeping track of when they took their doses. The medication administration log will include instructions on timing of the dose, what to do if a dose is missed and how to store the medication. If a dose is missed, they should take the dose as soon as it is remembered, but only one dose per day. Participants will be instructed to take their medication in the morning each day at the same time, preferably early in the morning. LDV/SOF FDC can be taken with or without food. Additionally, participants will be given adherence tips, such as setting an alarm on their phone, linking medication taking to another daily activity, etc. Participants will be instructed to bring any unused medication to their next visit and to bring their dose with them to the PK1, PK2 and PK3 Visits. Participants will be instructed to call the study staff immediately if they run out of medication or if they lose their study medication. In this case, all possible efforts will be made to get the participant study medication as soon as possible.

6.3 Supply and Accountability

6.3.1 Supply

Gilead Sciences will manufacture the study medication under Good Manufacturing Practices (GMP) and will package, label and ship all of the study medication directly to the PoR at Magee-Womens Hospital.

6.3.2 Storage and Dispensing

LDV/SOF FDC should be stored at room temperature below 30°C within the main pharmacy at Magee-Womens Hospital[38]. The PoR will maintain documentation of temperature in the area where the study medication is stored. Study medications will be dispensed from the pharmacy in a small bottle containing a quantity sufficient until the next study visit with three additional doses in the case of a lost dose or missed study visit. Study medications will be dispensed from the pharmacy only upon receipt of a written prescription from an authorized prescriber. The study medication will be dispensed to the study staff, then subsequently provided directly to the study participant in the research clinic.

6.3.3 Accountability

The PoR will maintain complete accountability records of all study LDV/SOF FDC received and dispensed. All unused study medications will be returned to Gilead Sciences or destroyed as instructed by Gilead after the study is complete.

6.3.4 Retrieval of Study Medication

The participants will be instructed to return any unused medication at each visit. If the participant fails to return unused medication at a scheduled visit, alternate arrangements will be made to obtain the unused medication from the participant (i.e. at an unscheduled visit). Unused medication will be accounted for by the clinician, documented in the participant's research record and then returned to the PoR.

6.4 Concomitant Medications

Enrolled study participants may use non-prohibited concomitant medications during study participation. All concomitant medications reported throughout the course of the study will be recorded on case report forms designated for that purpose. All prescription medications, over-the-counter preparations, vitamins, nutritional supplements, and herbal preparations will be recorded on forms for concomitant medications. Participants are prohibited from using medication contraindicated for concomitant use with Ledipasvir and Sofosbuvir as described in the package insert[38] Each reported concomitant medication will be reviewed to ensure the participant is not using prohibited medications (i.e. St. John's wort, rosuvastatin) as referenced in the Harvoni package insert. At each visit, participants will be asked if they have initiated any new medications or changed any reported medications. If the participant reports using acid suppressing medications, specific dosing instructions will be reviewed regarding correct timing of acid suppressing medication use according to the Harvoni package insert.

7 STUDY PROCEDURES

An overview of the study visit and evaluations schedule is presented in Appendix 1. Any clinical or laboratory information collected as a part of the participant's routine clinical care occurring on the same day as the study visit does not need to be repeated and can be collected from the participant's medical record.

7.1 Pre-screening

With IRB approval, study staff will pre-screen records to identify potential participants by evaluating minimum criteria (i.e. age, gestation age, Hepatitis C status). Clinic staff will be informed of the study and will be asked to provide an IRB approved ad/written material to potential participants or will assess interest. If the participant agrees, research staff can provide additional information by reviewing an IRB approved

screening script, which will include a brief overview of the study and minimum eligibility questions. Women who are interested and qualify based on the screening script may schedule a Screening Visit if interested. Process information (e.g., number of potential participants contacted, number presumptively eligible) may be recorded and stored at the study site in the absence of written informed consent from potential participants, provided the information is collected in such a manner that it cannot be linked to participant identifiers. Procedures and documentation will comply with the University of Pittsburgh IRB requirements. IRB approved materials (i.e. ads, flyers videos, and social media posts) may also be used (i.e. hung throughout the hospital; displayed on electronic boards; emailed to providers or appropriate support or community groups).

7.2 Visit 1- Screening (V1)

Screening can take place anytime within the Screening gestational age window (14+0 to 22+6 weeks with gestational age being determined by available clinical information at the Screening visit). Multiple visits may be conducted to complete all required screening procedures, if necessary. Written informed consent will be obtained before any screening procedures are initiated. Participants will be encouraged to speak with their primary care provider to determine whether to initiate hepatitis C treatment during pregnancy before deciding whether to participate in the study. For participants who subsequently do not meet the eligibility criteria, screening will be discontinued once ineligibility is determined. Any exclusionary laboratory values can be repeated at a later date within the Screening window. If the repeated laboratory values meet inclusion criteria then the participant can be enrolled. If there is concern that the participant's health status has changed between the enrollment visit and the screening visit or if there is concern by the study investigators that the participant might not remain eligible, the screening laboratories can be repeated prior to enrollment.

Table 3: Screening Visit (V1)

Visit 1- Screening Visit	
**Component**	**Procedures**
**Administrative and Regulatory**	• Review and obtain written informed consent<br>• ICF Comprehension Assessment<br>• Assign participant ID (PTID)<br>• Sign appropriate medical record releases (i.e. to obtain records from the Pregnancy Recovery Center; outside records as necessary)<br>• Collect locator information<br>• Collect demographic information<br>• Visit Questionnaire<br>• Assess eligibility<br>• Provide reimbursement for study visit<br>• Schedule next visit
**Clinical**	• Collect medical history & review and print medical/prenatal records including documentation of chronic Hepatitis C<br>• Review and obtain documentation of previous liver biopsy or liver imaging<br>• Collect concomitant medications<br>• Pre/post-test HIV counseling<br>• Perform full physical examination<br>• Assess fetal heart tones<br>• Confirm gestational dating (order ultrasound if necessary)

**Laboratory**	• Collect blood<br>– Complete blood count (CBC) with differential and platelets<br>– Creatinine<br>– HIV-1 serology<br>– Hepatitis B Virus testing (HBsAg, anti-HBc, Anti-HBs)<br>– Coagulation (PT/INR)<br>– HCV RNA viral load<br>– HCV genotype<br>– Hepatic function panel (AST, ALT, albumin, total and direct bilirubin, and alkaline phosphatase)<br>– Lipase<br>– Creatine kinase

7.3 Visit 2- Enrollment (V2)

The following procedures will occur at the Enrollment visit (V2). During the enrollment visit the participant will be given instructions on using and begin taking the study medication.

Table 4: Enrollment (V2)

Enrollment Visit- Visit 2 (V2)	
**Component**	**Procedures**
**Administrative and Regulatory**	• Confirm eligibility<br>• Review/update locator information<br>• Visit Questionnaire<br>• Provide reimbursement for study visit<br>• Schedule next visit
**Behavioral**	• Conduct in depth interview<br>• Provide counseling<br>– Protocol adherence<br>– Study medication use/adherence
**Clinical**	• Review/update medical history<br>• Review/update concomitant medications<br>• Document pre-existing conditions<br>• Perform full physical examination<br>• Assess fetal heart tones<br>• Provide Screening test results
**Medications**	• Participants will receive study medication (supply enough for next visit plus 3 extra doses)<br>• Participants will receive instructions for use daily and for PK visits<br>• Participants will receive medication adherence log
**Laboratory**	• HCV RNA viral load

7.4 Follow-up Visits

7.4.1 Visits 3-5 (PK 1, PK2, PK3)

The following procedures will occur on at the following gestational ages:
1) **PK1:** between 25 + 0 and 26 + 6 weeks' gestation (after at least 10 days of therapy)
2) **PK2:** 29 + 0 and 30 + 6 weeks' gestation
3) **PK3:** 33 + 0 and 34 + 6 weeks' gestation

The PK visits will be scheduled as **early** as possible within the gestational age window to allow for visits that require rescheduling.

Table 5: Follow-up Visits (PK1, PK2, and PK3)

Follow-up Visits 3-5 (PK1, PK2, PK3)	
**Component**	**Procedures**
**Administrative and Regulatory**	• Review/update locator information<br>• Visit questionnaire<br>• Provide reimbursement for study visit<br>• Record/update AEs<br>• Schedule next visit (PK 2, PK 3, End of Treatment)
**Behavioral**	• Adherence assessment<br>• Provide modified counseling<br>– Protocol adherence<br>– Instructing participants to contact study staff when in labor/being admitted to labor and delivery<br>– Study medication use/adherence
**Clinical**	• Review/update medical history<br>• Review/update concomitant medications<br>• Perform modified physical examination<br>• Assess fetal heart tones<br>• Provide available test results (if applicable)<br>• Administer timed dose of study medication<br>• Provide study medication with sufficient supply until next visit (PK1 and PK2) or until completion of treatment course (PK3) with 3 additional doses<br>• Adherence assessment
**Laboratory**	• Collect blood<br>– Intensive PK sampling (all visits)<br>– Protein binding assay (all visits)<br>PRIOR TO VISIT DOSING:<br>– CBC with differential and platelets (PK1 and PK2)<br>– Hepatic function panel (AST, ALT, albumin, total and direct bilirubin, alkaline phosphatase) (PK1 and PK2)<br>– PT/INR (PK1 and PK2)<br>– Creatinine (PK1 and PK2)<br>– Lipase (PK1 and PK2)<br>– Creatine kinase (PK1 and PK2)<br>– HCV RNA viral load (PK1 and PK2)<br>– HCV Resistance Testing*

*as indicated per protocol section 9.3

LDV and SOF PK Evaluation

Participants will be instructed not to take their daily dose at home but rather to bring their dose of SOF/LDV FDC to their visit for a timed administration. Alternately, a dose will be dispensed by the PoR for the PK visits. Participants will be instructed

about dietary restrictions for these visits as outlined in section 7.6. Participants will have their first PK plasma drawn before the time of their dose of SOF/LDV FDC. The dose at each PK visit will be timed and blood samples will be collected at each PK visit as follows (relative to the timed dose): pre-dose, 0.5, 1, 2, 3, 4, 5, 8 and 12 hours. An intravenous catheter will be placed prior to the pre-dose draw and will be in place throughout the PK visits. A saline solution will be used at a slow drip to attempt to keep the vein patent. In the event that the catheter fails, participants may have another IV inserted or individual venipuncture(s) to attempt to collect all PK samples. If a PK sample(s) cannot be obtained, then the participant will remain evaluable, if at least one sample is obtained at each PK visit. For further details, see section 7.6 Pharmacokinetics.

7.4.2 End of Treatment Visit (V6)

The following procedures will occur at the End of Treatment visit. This visit will be scheduled within 7 days of completion of the 12 week course of study medication. The purpose of this visit will be to collect any remaining study medication and to collect and follow up on any adverse events. Participants will also be reminded to call the research staff when they present to the hospital for delivery.

Table 6: End of Treatment Visit

**End of Treatment Visit**	
**Component**	**Procedures**
**Administrative and Regulatory**	• Review/update locator information<br>• Visit Questionnaire<br>• Provide reimbursement<br>• Record/update AEs
**Behavioral**	• Protocol adherence counseling, including delivery visit instructions<br>• Collect remaining study medication<br>• Collect medication administration log<br>• Conduct in depth interview
**Clinical**	• Review/update medical history<br>• Review/update concomitant medications<br>• Perform modified physical examination<br>• Provide available test results if available<br>• Assess fetal heart tones (if applicable)
**Laboratory**	• HCV RNA viral load<br>• HCV resistance testing*

*as clinically indicated

7.4.3 Delivery Visit (in person visit or chart review)

Participants will be instructed to contact the study staff when they are in labor/admitted to labor and delivery, regardless of delivery location. The Delivery Visit will occur during admission for participants delivering at Magee-Womens Hospital and will include maternal and cord blood samples. The date of the Delivery Visit for participants who deliver at Magee will be the date the study staff makes the first contact with the participant during her delivery admission, regardless if procedures occur over more than one day. Participants who deliver at an outside location will be contacted by phone as close to delivery as possible to complete the applicable Delivery Visit study procedures (i.e. questionnaire, update locator

information, update medical history). The Delivery Visit date for these participants will be the date of the completed phone call. All participants, regardless of delivery location will undergo a chart review to obtain delivery outcomes/variables. A copy of the delivery records will be placed in the participant's research chart. In the event that the participant delivers prematurely at Magee-Womens Hospital of UPMC and is still taking study medication, then procedures from previously scheduled visits (V2-V6) may be performed, excluding intensive PK sampling. Rather than intensive PK sampling, a single maternal blood sample will be taken as close to the time of delivery as possible and cord blood will be collected for LDV/SOF PK analysis and the protein binding assay.

Table 7: Delivery Visit

Delivery Visit	
**Component**	**Procedures**
**Administrative and Regulatory**	• Review/update locator information<br>• Visit Questionnaire<br>• Provide reimbursement, as applicable<br>• Record/update AEs<br>• Schedule next visit
**Behavioral**	• Protocol adherence counseling<br>• Study medication use/adherence (if still on study medication)
**Clinical**	• Review/update medical history<br>• Review/update concomitant medications<br>• Provide available test results if available<br>• Perform modified physical examination (or obtain from medical record)<br>• Dispense study medication to complete 12 week course (if still on study medication)*
**Medical Record Review for Delivery Outcomes**	• Type of pregnancy outcome<br>• Gestational age at pregnancy outcome<br>• Method of calculation for gestational age at pregnancy outcome<br>• If delivery, type of delivery (e.g., vaginal, vaginal forceps-assisted, vaginal vacuum-assisted, cesarean section)<br>• Complications related to pregnancy outcome<br>– Delivery complications (e.g., intrapartum and/or postpartum hemorrhage, non-reassuring fetal status, chorioamnionitis)<br>– Other complications not related to a delivery
**Baseline Infant Information****	• Sex<br>• Weight<br>• Length<br>• Head circumference<br>• Apgar scores<br>• Medical history (e.g., sepsis, respiratory distress, any abnormalities noted on infant exam)<br>• Medication history
**Laboratory*****	• Maternal HCV RNA viral load<br>• Cord blood collection for HCV RNA viral load<br>• Cord blood collection for PK analysis (if still on study medication)<br>• Maternal plasma sample for PK analysis (if still on study medication)<br>• Maternal protein binding assay (if still on study medication)<br>• Cord blood protein binding assay (if still on study medication)

*Unless participant intends to breastfeed, in which case study medication will be discontinued
**All Baseline Infant Information may be extracted from the infant medical record as available
***Laboratory tests performed as applicable for participants who deliver at Magee-Womens Hospital

7.4.4 Post Treatment (V7) Visit

The following procedures will occur at the Post Treatment visit (V7), which should be scheduled 12 weeks ± 4 days from the last dose of study medication. The V7 visit can occur simultaneously with the 8 week Follow-up Visit for the infant (iV1). This is the last scheduled clinic visit for the maternal participant.

Table 7: Post Treatment (V7)

Visit 7 (V7) Post Treatment Visit	
**Component**	**Procedures**
**Administrative and Regulatory**	• Review/update locator information<br>• Visit questionnaire<br>• Provide reimbursement<br>• Record/update AEs<br>•
**Behavioral**	• Protocol adherence counseling
**Clinical**	• Review/update medical history<br>• Review/update concomitant medications<br>• Perform modified physical examination<br>• Provide available test results
**Laboratory**	• Collect blood<br>– CBC with platelets and differential<br>– Hepatic function panel (AST, ALT, albumin, total and direct bilirubin, alkaline phosphatase)<br>– PT/INR<br>– Creatinine<br>– Lipase<br>– Creatine kinase<br>– HCV RNA viral load*<br>– HCV resistance testing (only indicated if HCV RNA is detectable)

*If HCV RNA is detectable, the participant would be asked to return to have HCV resistance testing done, and if participant was still on study medication at time of delivery and study medication was discontinued at delivery visit because the participant is breastfeeding.

7.4.5 Infant Follow-Up Visits (iV1, iV2, iV3)

After delivery, infants will be seen at the following time points:
iV1: 8 weeks ± 6 weeks
iV2: 6 months ± 2 weeks
iV3: 12 months ± 2 weeks

The following procedures will occur at the Infant Follow-Up Visits (iV1, iV2, iV3). If blood sampling is not possible, then the results can be collected from the infant'smedical record if available. Results of physical and developmental exams

will be shared with the mother, and if necessary with the infant's routine healthcare provider.

Table 8: Infant Follow-Up Visits (iV1, iV2, iV3)

Visit iV1, iV2, iV3 Infant Follow-Up Visits	
**Component**	**Procedures**
**Administrative and Regulatory**	• Review/update locator information<br>• Review infant demographics (iV1)<br>• Sign release(s) for medical records for infant<br>• Provide reimbursement<br>• Schedule next visit (iV1, iV2)
**Clinical**	• Review/update medical history<br>• Review/update concomitant medications<br>• Growth assessment including weight, length, and head circumference<br>• Perform age appropriate physical examination<br>• Bayley's Developmental Exam (iV2 and iV3 only)<br>• Provide available test results
**Laboratory**	• Collect infant blood<br>– HCV RNA (iV1, iV2, and as applicable at iV3)*<br>– HCV resistance testing (if HCV RNA is detectable)

*If HCV RNA is detectable at iV1 or iV2, the infant would need to return to have HCV resistance testing done and would also have an HCV RNA performed at iV3. If HCV RNA is non-detectable/negative at both iV1 and iV2, no additional samples are needed.

7.5 Follow-up Procedures for Participants Who Permanently Discontinue Study Medication

Participants will be permanently discontinued for significant laboratory abnormalities or adverse events as outlined in section 9.3 and 9.4, if they miss greater one dose per week (averaged over the duration of the study), or if they choose to discontinue the study medication. Participants who are permanently discontinued from the study will be instructed to return the study medication and study medication log. All protocol-specified study procedures will continue for safety except the following:

- Provision of study medication
- Provision of medication use adherence counseling

The following procedures will be performed at the visit in which study medication use is permanently discontinued:

- Collection of blood for PK analysis (single time point)
- HCV resistance testing
- Viral load as needed (i.e. participant withdraws, investigator opinion)
- Medical history review
- Concomitant medication review
- Brief physical examination including vital signs, if indicated.

The participant will be asked to continue in the study and complete all remaining scheduled maternal and infant visits per protocol for safety.

7.5.1 Interim Visits

Interim visits may be performed at any time during the study. Study procedures may be repeated at interim visits as deemed clinically indicated. All interim contacts and visits will be documented in participants' study records and on applicable CRFs.

7.6 Pharmacokinetics

All enrolled participants will undergo intensive serum PK specimen collection procedures. These collections will occur at study visits as described in the table 9.

Table 9: SOF and LDV PK Sampling

Time since LDV/SOF*	Plasma Sample	Meal	LDV/SOF PK sample (mL)	Protein Binding (mL)	Total blood (mL)
predose	+	+	4	0	4
30 mins	+		4	0	4
1 hour	+		4	0	4
2 hours	+		4	12	16
3 hours	+		4	0	4

4 hours	+	+	4	0	4
5 hours	+		4	0	4
8 hours	+	+	4	0	4
12 hours	+		4	0	4

*Time listed is the target time for PK sample. If a sample is collected prior to next scheduled PK sample it will not be considered a protocol deviation. Only missed samples will be considered protocol deviations.

Participants will be instructed to bring their daily dose of LDV/SOF FTC to the visits requiring intensive PK sampling (PK1, PK2, and PK3 visits). If they do not bring their dose, then a dose of study medication can be dispensed from the pharmacy/PoR. Intravenous catheters will be placed upon arrival and a pre-dose sample will be collected. Study drug will be administered as close as possible to the participant's normal dose time and within 5 minutes of completing and within 30 minutes of initiating a standardized moderate-fat-calorie breakfast (~600 calories and 25 to 30% fat) following an overnight fast (no food or liquids, except water, for at least 10 hours). Participants will be restricted from food intake until after collection of the 4 hour blood draw and restricted from water consumption 1 hour before and 2 hours after dosing, except for approximately 240 mL of water given with study drug and standardized meal. Whole blood will be collected into appropriate sized Vacutainer® Plus plastic sterile tube(s) with K_2EDTA (spray dried) additive. Immediately after collection, the collection tube will be inverted 8 to 10 times to allow mixing with the anticoagulant. Further processing instructions can be found in the Specimen Collection and Procedures Manual Version 1.0.

7.7 Adherence Counseling and Assessment

Adherence counseling to the study protocol will be performed at each study visit. Additionally, investigators may use text messaging (daily or weekly), follow-up phone calls and meeting study participants at their regular clinic visits in order to improve adherence to the study medication. All options will be included in the informed consent document and will be tailored to the participant's needs. At the follow-up visits (PK1, PK2, and PK3), all remaining medication will be counted, recorded and returned to the pharmacy. Study participants will be given a medication administration log upon enrollment to assist in study medication adherence. Each log will have instructions on how and when to take the medication. We will recommend that participants take the study medication between 8:00am and 9:00am daily. If a participant forgets to take a dose, she should take the missed dose as soon as she remembers but should never take more than one tablet of LDV/SOF FDC per day. Directions on how to store the medication as well as what to do if the medication is lost or stolen will be included on the medication administration log. Participants will be asked to bring the medication administration log to the next study visit for review. Medication administration logs will be collected at the next visit and a new log will be given with each bottle/refill of study medication. Adherence counseling will be performed at the

following visits: enrollment, PK1, PK2, and PK3 and at delivery has not completed the 12 weeks of study medication and is not breastfeeding).

7.8 In Depth Interviews and Qualitative Analysis

At the Enrollment visit and the End of Treatment visit a trained interviewer from the Qual EASE team (CHRC Data Center, University of Pittsburgh) will conduct an in-depth interview about Hepatitis C infection, risks of perinatal transmission, concerns about treatment during pregnancy and drug research during pregnancy. The interviewer will use the HIP Script to conduct the interview. All interviews will be recorded and transcribed verbatim. The original recordings will be destroyed after transcription. Following transcription, the Qual EASE team will develop a qualitative codebook using an editing approach to ensure that all relevant topics and themes are represented. Two trained qualitative coders from Qual EASE will then be trained in the codebook. A primary coder will code all 15 interviews in the pre- and post-treatment interview groups (for a total of 30 coded transcripts), and a secondary coder will code 10 interviews in the pre- and post-treatment interview groups (for a total of 20 coded transcripts). Coding will be completed using Atlas.ti software, which will help to determine the frequency and prevalence of the topics and themes which were discussed. Cohen's Kappa scores will be calculated to determine intercoder reliability. Thematic analysis will then be completed by Qual EASE staff using constant comparative method.

7.9 Clinical Evaluations and Procedures

Physical exams will include the following assessments:

- General appearance
- Weight
- Vital signs
  - Temperature
  - Pulse
  - Blood pressure
  - Respirations
- Height *
- Abdomen*
- Head, Eye, Ear, Nose and Throat (HEENT) Examination*
- Lymph nodes*
- Neck*
- Heart*
- Lungs*
- Extremities*
- Skin*
- Neurological*

*may be omitted after the Enrollment Visit

Additional clinical assessments may be performed at the discretion of the examining clinician in response to symptoms or illnesses present at the time of the exam.

7.10 Laboratory Evaluations

<u>Local Laboratory (UPMC Clinical Laboratory)</u>

- Blood
    - HIV serology
    - Hepatitis B Virus testing
        - HBsAg
        - Anti-HBc
        - Anti-HBs
    - PT/INR
    - Lipase
    - Creatine kinase
    - HCV RNA viral load: Maternal and Infant (Abbott RealTime HCV assay)
    - Hepatitis C genotype
    - CBC with differential and platelets
    - Creatinine
    - Hepatic function panel
        - AST
        - ALT
        - Albumin
        - Total and direct bilirubin
        - Alkaline phosphatase

<u>Outside Laboratory</u>

- Blood
    - Blood PK for SOF and LDF (Sample Team Delaware)
    - Protein Binding Assay (QPS, LLC)
    - HCV Resistance Testing (Quest Lab)

7.11 Specimen Collection and Processing

The study site will adhere to the standards of good clinical laboratory practice and standard operating procedures for proper collection, processing, labeling, transport, and storage of specimens. Specimens for LDV/SOF PK analysis and protein binding will be transported from the Clinical Research site to the Magee-Womens Research Institute Microbiology Laboratory for storage until they are shipped to the appropriate laboratory for processing and analysis. The samples will be shipped when arranged with the respective laboratories listed for PK analysis and protein binding.

7.12 Biohazard Containment

As the transmission of hepatitis C and other blood-borne pathogens can occur through contact with contaminated needles, blood, and blood medications, appropriate blood and secretion precautions will be employed by all personnel during blood draws and transport, shipping and handling of all specimens for this study as recommended by the CDC and National Institutes of Health (NIH). All biological specimens will be shipped using packaging mandated by Code of Federal Regulations (CFR) 42 Part 72. All dangerous goods materials, including diagnostic specimens and infectious substances, must be shipped according to instructions detailed in the International Air Transport Association (IATA) Dangerous Goods Regulations. Biohazardous waste will be contained according to institutional, transportation/carrier, and all other applicable regulations.

8 ASSESSMENT OF SAFETY

8.1 Safety Monitoring

Study investigators are responsible for continuous close safety monitoring of all study participants, and for notifying Gilead Sciences if unexpected and/or serious events occur. An independent physician safety monitor who is not otherwise involved in the study will review the safety data once per month or more frequently as needed throughout the period of study implementation, discuss study medication management, and address any potential safety concerns.

8.2 Clinical Data and Safety Review

The clinical research investigators are responsible for the initial evaluation and reporting of safety information at the participant level to the Protocol Chair. The Protocol Chair is responsible for alerting Gilead Sciences if unexpected concerns arise. Additional reviews may be conducted at each of these levels as dictated by the occurrence of certain events.

The Reproductive Infectious Disease Data Management team will review incoming safety data on an ongoing basis. Events identified as questionable, inconsistent, or unexplained will be queried for verification. The clinical investigator and the independent safety monitor will meet approximately every month to review clinical data reports. The content, format and frequency of the clinical data reports will be agreed upon by the independent safety monitor, Gilead Science and the study investigators in advance of study implementation. In addition to the routine safety data reviews, the independent safety monitor and the study investigators will convene on an ad hoc basis to make decisions regarding the handling of any significant safety concerns. If necessary, external experts representing expertise in the fields of pregnancy, infectious diseases, hepatology and medical ethics may be invited to join the safety review. A recommendation to pause or stop the trial may be made at this

time or at any such time that the safety review team agrees that an unacceptable type and/or frequency of AEs has been observed.

8.3 Adverse Events Definitions and Reporting Requirements

8.3.1 Adverse Events

An AE is defined as any untoward medical occurrence in a clinical research participant administered an investigational medication and which does not necessarily have a causal relationship with the investigational medication. As such, an AE can be an unfavorable or unintended sign (including an abnormal laboratory finding, for example), symptom or disease temporally associated with the use of an investigational medication, whether or not considered related to the medication. The term "investigational medication" for this study refers to the study medication.

Study participants will be provided instructions for contacting the study site to report any untoward medical occurrences they may experience throughout their participation in the study. Participants receiving prenatal care and/or delivering at Magee-Womens Hospital will be instructed to come to Magee-Womens Hospital if they experience any adverse events requiring evaluation. Participants will be seen by a physician investigator/study clinician. If the participant is not receiving care at Magee-Womens Hospital, then they will be instructed to seek medical care with their local primary care physician/obstetrical team. Close communication with the participant and the primary care provider/obstetrical team will be maintained. Participants may have an unscheduled visit with the physician investigator/study clinicians as necessary or desired. In cases of potentially life-threatening events, participants will be instructed to seek immediate emergency care. With appropriate permission of the participant, whenever possible, records from all non-study medical providers related to untoward medical occurrences will be obtained and required data elements will be abstracted and recorded on study CRFs. All participants reporting an untoward medical occurrence will be followed clinically until the occurrence resolves (returns to baseline) or stabilizes.

Study site staff will document all AEs reported by or observed in enrolled study participants regardless of severity and presumed relationship to investigational medication, including gradable laboratory findings. AE severity will be graded per the DAIDS Table for Grading Adult and Pediatric Adverse Events, Version 1.0, December 2004 (Clarification dated August 2009).

8.3.2 Serious Adverse Events

An SAE will be defined as an AE that:

- Results in death
- Is life-threatening

- Requires inpatient hospitalization or prolongation of existing hospitalization
- Results in persistent or significant disability/incapacity
- Is a congenital anomaly/birth defect
- Is an important medical event that may not result in death, be immediately life-threatening, or require hospitalization but may jeopardize the participant or require intervention to prevent one of the outcomes listed in the definition above.

8.3.3 Adverse Event Relationship to Study Medication

Relatedness is an assessment made by the physician investigator of whether or not the event is related to the study agent.

- *Related:* There is a reasonable possibility that the AE may be related to the study agent(s)
- *Not Related:* There is not a reasonable possibility that the AE is related to the study agent(s)

8.4 Expedited Adverse Event Reporting Requirements

8.4.1 Reporting Requirements for this Study

All adverse events will be reported to Gilead Sciences on a monthly basis. All serious adverse events will be reported to the University of Pittsburgh Institutional Review Board, the FDA and Gilead Sciences according to the University of Pittsburgh IRB's reporting guidelines.

8.4.2 Grading Severity of Events

The most current Division of AIDS Table for Grading the Severity of Adult and Pediatric Adverse Events (DAIDS AE Grading Table) is available on the RSC website at http://rsc.tech-res.com/safetyandpharmacovigilance/.

8.5 Regulatory Requirements

Information on all reported AEs will be included in reports to the FDA and other applicable government and regulatory authorities. The Protocol Chair will submit AE information in accordance with the requirements of the University of Pittsburgh IRB.

8.6 Social Harms Reporting

Although every effort will be made to protect participant privacy and confidentiality, it is possible that participants' involvement in the study could become known to others and that social harms may result. Social harms that are judged by the study investigators to be serious or unexpected will be reported to Gilead Sciences and to the University of Pittsburgh IRB.

9 CLINICAL MANAGEMENT

Guidelines for clinical management and permanent discontinuation of study medication are outlined in this section. In general, the physician investigators will only discontinue study medication in consultation with the independent physician safety monitor if they feel that the risk of study medication continuation outweighs the benefits of study medication continuation. The physician investigators will document all permanent discontinuations on applicable CRFs.

9.1 Grading System

AE severity grading is described in Section 8.3.1.

9.2 Dose Modification Instructions

No dose modifications will be permitted in this study.

9.3 General Criteria for Permanent Discontinuation of Study Medication

A participant will be permanently discontinued from medication use by the physician investigators for any of the following reasons, according the HCV treatment guidelines[23]:

- 5-fold or greater increase in ALT or AST at the PK1 or PK2 visit compared to baseline result (Screening visit), confirmed by immediate repeat testing
- Any increase of ALT or AST of less than 5-fold from baseline (Screening visit) at the PK1 or PK2 visit that is accompanied by any weakness, nausea, vomiting, or jaundice
- 3-fold or greater increase in ALT or AST accompanied by bilirubin >2x the upper limit of normal, confirmed on immediate repeat testing
- Increase HCV RNA viral load by greater than 10-fold at PK2 visit (6-8 weeks after starting treatment)
- Participant is unable or unwilling to comply with required study procedures
- If the participant delivers prematurely (and is still on study medication) and desires to breastfeed
- Participant might be put at undue risk to their safety and well-being by continuing medication use, according to the judgment of the study investigators. The study investigators will consult with the independent safety physician prior to all study medication discontinuation instituted for this reason.

Any time a participant is permanently discontinued from study medication (prior to completion of the 12 week course of study medication), HCV resistance testing will be performed.

9.4 Permanent Discontinuation in Response to Adverse Events

Grade 1 or 2

In general, a participant who develops a Grade 1 or 2 AE as defined by the DAIDS Table for Grading Adult and Pediatric Adverse Events, Version 1.0, December 2004 (Clarification dated August 2009) regardless of relationship to study medication will continue study medication use.

Grade 3

For participants who develop a Grade 3 AE as defined by the DAIDS Table for Grading Adult and Pediatric Adverse Events, Version 1.0, December 2004 (Clarification dated August 2009) that is judged by the study investigators to be unrelated to study medication, the study medication will continue. The study medication must be permanently discontinued for participants who develop a Grade 3 AE judged by a physician investigators to be related to the study medication.

Grade 4

If a participant develops a Grade 4 AE as defined by the DAIDS Table for Grading Adult and Pediatric Adverse Events, Version 1.0, December 2004 (Clarification dated August 2009) and the AE is determined to be related to study medication, then study medication must be permanently discontinued.

Any time a participant is permanently discontinued from study medication (prior to completion of the 12 week course of study medication), HCV resistance testing will be performed.

9.5 HIV-1 Infection

Participants who are positive for HIV (known or positive at Screening) will not be eligible to participate. If HIV seroconversion occurs during the time of study medication use, the study medication will continue.

9.6 Criteria for Early Termination of Study Participation

Participants may voluntarily withdraw from the study for any reason at any time. The study investigators also may withdraw participants from the study to protect their safety and/or if they are unwilling or unable to comply with required study procedures. Participants also may be withdrawn if Gilead Sciences, government or regulatory authorities, including the FDA and Office for Human Research Protections (OHRP), or site IRBs/ECs terminate the study prior to its planned end date. Detailed reason with the withdrawal of a participant will be documented in the research record. Every

reasonable effort will be made to continue to follow the participant as scheduled for safety

10 STATISTICAL CONSIDERATIONS

10.1 Overview and Summary of Design

This is a Phase 1, single-arm, open label pilot study of treatment for chronic HCV infection during pregnancy in 15 HCV (genotype 1, 4, 5, and 6)-infected pregnant women with a 12-week course of LDV/SOF FDC.

10.2 Study Endpoints

Pregnant women meeting all of the criteria in section 5.2 and 5.3 will be enrolled into the study and followed prospectively through 12 weeks after the last dose of study medication. Infants will be enrolled upon delivery and followed prospectively through approximately one year of age.

10.2.1 Primary Endpoints: Maternal

The primary endpoints of the study are primary PK parameters (specifically AUC_{tau}, C_{max}, and C_{tau}) of LDV, SOF, and GS-331007 assessed at 3 gestational age time points:

1) Late second trimester between 25 + 0 and 26 + 6 weeks' gestation (after at least 10 days of therapy

2) Early third trimester between 29 + 0 and 30 + 6 weeks' gestation and

3) Late third trimester between 33 + 0 and 34 + 6 weeks' gestation.

10.2.2 Secondary Endpoints: Maternal

The secondary endpoints of the study are:

1) Secondary PK endpoints of LDV, SOF and GS-331007:
    a. Total clearance (CL/F)
    b. Protein binding
    c. Unbound clearance
2) Maternal sustained virologic response after 12 weeks of therapy (SVR12)
3) Adverse events including clinical and laboratory changes
4) Pregnancy outcomes including the following:
    a. delivery prior to 37 completed weeks of gestation
    b. stillbirth or intrauterine fetal demise
    c. intrapartum hemorrhage
    d. postpartum hemorrhage
    e. non-reassuring fetal status
    f. chorioamnionitis

g. hypertensive disorders of pregnancy
h. gestational diabetes
i. intrauterine growth restriction

Secondary Endpoints: Infant

Consistent with the secondary study objectives, the following secondary endpoint will be assessed for infants:

- Major malformations, defined as structural abnormalities with surgical, medical, or cosmetic importance.
- Weight, length, and head circumference at birth, 8 weeks, six months and 12 months.
- Neurodevelopmental assessments at 6 months and 12 months as measured by Bayley Neurodevelopment Screening Test.
- Perinatal HCV transmission assessed by presence of HCV RNA at 8 weeks, six months or 12 months.

10.3 Sample Size

To evaluate the primary objective, we estimate a desired sample size of 15 participants. We anticipate that we are more likely to see a change in sofobuvir metabolism during pregnancy because of its urinary excretion and increased renal blood flow during pregnancy. The sample size is based on GS-3311007 because of its longer half-life, when compared to sofosbuvir. This samples size provides an estimated power of 0.80 to detect a 35% change in the AUC_{tau} of the primary sofobuvir metabolite GS-3311007 and the reported interpatient variability of 30.7% of GS-3311007 AUC_{tau}[41]. Additionally, this sample size will also allow for detection of a 40% change in AUC_{tau} of GS-3311007 if the interpatient variability is increased to 40%. Similarly, this samples size provides an estimated power of 0.80 to detect a 35% change in the AUC_{tau} of ledipasvir and the reported interpatient variability of 33.9% of LDV AUC_{tau}[42].

Any participant that discontinues the study before the PK3 visit is completed or if a participant reports missing more than 1 dose per week averaged over the duration of the study then they will be replaced by recruiting another participant into the study.

Table 10: Sample Size Calculation and Power Analysis

Sample size	Alpha Level	Expected Effect on GS-331007 AUC_{tau}	Coefficient of Variation (CV) GS-331007	Power Estimated
15	0.05	40%	30.7%	95%
15	0.05	35%	30.7%	90%
15	0.05	45%	40%	87%
	Alpha	AUC_{tau} LDV	CV LDV	Power
15	0.05	35%	33.9%	80%
15	0.05	40%	40%	87%

10.4 Participant Accrual and Retention

Pregnant and chronically HCV-infected women who meet the criteria outlined in Section 5 and are interested in participating, will be enrolled into this study. Once a participant is enrolled, the study staff will make every reasonable effort to retain her for the entire study period. A maximum of 7% (one woman and child) loss-to-follow-up will be targeted.

10.5 Data Analysis

10.5.1 Analysis of SOF and LDV concentrations

Between-group comparisons will evaluate changes in SOF and LDV PK parameters between the historical controls (nonpregnant women of reproductive age) compared to the 15 pregnant women stratified by gestational age. Area under the time concentration cure over a dosing interval (AUC_{tau}), maximum concentration (C_{max}), and minimum concentration (C_{tau}) will be calculated by gestational age. The PK parameters of the pregnant women at different gestational ages will be compared to the historical control group by calculating the geometric mean ratios and 90%CIs. Drug concentrations will be summarized descriptively (mean, median, minimum, maximum, standard deviation) by gestational age.

10.5.2 Analysis of SVR12

Outside of pregnancy, 96% of patients treated with LDV/SOF for 12 weeks obtained an SVR12. In this pilot study, we estimate that at least 14 out of the 15 chronically HCV-infected pregnant women will have a SVR12 after treatment. Participants who do not complete the treatment course or report low adherence will be excluded from the analysis.

10.5.3 Analysis of Safety Endpoints

Rates of abnormal safety laboratory assessments will be compared to those reported in the literature on historic controls and rates of adverse pregnancy outcomes will be compared to historic HCV-infected pregnant women who have delivered at our institution. Statistical significance will be determined by chi-square test.

10.5.4 Data and Safety Monitoring Plan

No Data and Safety Monitoring Board oversight is planned for this study, however a Study Steering Committee comprised by the Protocol Chair, a second physician investigator from the University of Pittsburgh, a laboratory scientist from the University of Pittsburgh, the Data Management lead statistician and a representative from Gilead Sciences Clinical Research will provide oversight. Reviews of study progress, including rates of participant accrual, retention, completion of primary and main secondary endpoint assessments will take place approximately every 3

months, and as needed. At the time of these reviews, or at any other time, the Study Steering Committee may recommend that the study proceed as designed, proceed with design modifications, or be discontinued. An independent safety physician experienced with monitoring of clinical trials among pregnant women will conduct interim safety reviews on a monthly basis or more frequently at the request of the Study Steering Committee.

11 DATA HANDLING AND RECORDKEEPING

11.1 Data Management Responsibilities

Study CRFs will be developed by the study team in conjunction with data management.

11.2 Source Documents and Access to Source Data/Documents

The site will maintain source data/documents in accordance with current DAIDS policies. (http://rsc.tech-res.com/policiesandregulations/)

The study team will maintain, and store securely, complete, accurate and current study records throughout the study. In accordance with U.S. regulations regarding testing investigational medications, the study investigator will maintain all study documentation for at least two years following the date of marketing approval for the study medications being tested for the indication in which they were studied. If no marketing application is filed, or if the application is not approved, the records will be retained for two years after the investigation is discontinued and the US FDA is notified. For research involving children, records shall be retained at least until the pediatric subject reaches the age of 23.

Study records will be maintained on site for the entire period of study implementation.

11.3 Quality Control and Quality Assurance

The study site will conduct quality control and quality assurance procedures in accordance with site SOPs.

12 CLINICAL SITE MONITORING

As this is an investigator initiated IND study, monitoring will be performed by internal clinical study staff (i.e. QA/QC Reviewer), PoR, laboratory staff and local data management team. Internal reviews will include:

- Review informed consent forms, protocol procedures, and study documentation

- Assess compliance with the study protocol, Good Clinical Practices (GCP) guidelines, and applicable regulatory requirements (US and non-US), including CFR Title 45 Part 46 and Title 21 Parts 50, 56, and 312
- Perform source document verification to ensure the accuracy and completeness of study data
- Verify proper collection and storage of biological specimens
- Verify proper storage, dispensing, and accountability of investigational study medications

The Education and Compliance Office for Human Subject Research (ECO-HSR), Research Conduct and Compliance Office will also oversee study activities. The ECO-HSR has extensive experience in the auditing and monitoring of clinical investigations for compliance with GCP standards and IND commitments. To ensure appropriate institutional oversight of University-based IND applications, the ECO-HSR will periodically monitor the research oversight programs of IND Sponsors, which will include compliance of the Sponsor and Investigator with applicable FDA regulations, applicable University of Pittsburgh policies and the IRB-approved protocol and consent document. The frequency of these monitoring visits shall be determined by the ECO-HSR.

The study investigators also will allow inspection of all study-related documentation by authorized representatives of Gilead Sciences, FDA, OHRP, IRBs/ECs and other local and US regulatory authorities.

13 HUMAN SUBJECTS PROTECTIONS

Study investigators will make efforts to minimize risks to participants. Informed consent will be reviewed in detail with potential participants and all questions will be adequately answered prior to obtaining written informed consent. All eligibility criteria will be verified prior to initiation of investigational product. Recruitment will begin after receiving IRB approval and after the protocol has been submitted to the FDA. The study investigators will permit audits by the NIH, Gilead Sciences, the FDA, OHRP, IRB, and other local and US regulatory authorities or any of their appointed agents.

13.1 Institutional Review Boards

The study staff will ensure that the protocol, associated informed consent form, and study-related documents (such as participant education and recruitment materials) are reviewed and approved by the University of Pittsburgh IRB prior to starting the study. Any amendments to the protocol or informed consent will be approved by the University of Pittsburgh IRB prior to implementation.

13.2 Study Coordination

Catherine Chappell, MD MSc holds the Investigational New Drug (IND) application for this study. Assignment of all sponsor responsibilities for this study will be specified in a Clinical Trials Agreement (CTA) executed by the University of Pittsburgh and Gilead Sciences.

Close coordination between the study team is necessary to track recruitment, enrollment, retention, AEs and unanticipated problems and to address other issues that may arise in a timely manner. The study investigators and the independent safety physician will address issues related to study eligibility, AE management/reporting and unanticipated problems as needed to assure consistency. Rates of accrual, protocol adherence, retention, and AE incidence will be reported by data management and monitored closely by the team as well as the Study Steering Committee.

13.3 Risk Benefit Statement

13.3.1 Risks

<u>General/Maternal</u>

As with any research study, there may be adverse events or side effects for the maternal participant or the fetus/infant that are currently unknown and certain of these unknown risks could be permanent, severe or life-threatening.

There are no studies of LDV/SOF in pregnant women. There have been animal studies done in rats and rabbits as detailed in section 2.4.2. No effects on fetal development have been observed in rats and rabbits at the highest doses tested. However, animal reproductive studies are not always predictive of human response.

It is also not known if LDV/SOF is present in human breast milk. If the participant delivers while still on study medication and intends to breast feed, study medication will be discontinued and HCV resistance testing will be done at the next study visit.

There is a potential risk that the hepatitis virus may become resistant to LDV/SOF because the dose may not be adequate for treating pregnant women with hepatitis C or if the participant does not complete the entire 12 weeks of study medication. If resistance occurs, this may limit the choices of effective therapy after pregnancy for the participant and/or possibly her infant.

The following side effects have been associated with the use of oral LDV/SOF used daily for 12 weeks of duration in men and non-pregnant women:

	Percentage of Participants N=539
Fatigue	13%

Headache	14%
Nausea	7%
Diarrhea	3%
Insomnia	5%

There are risks of alterations in laboratory values with the use of this medication. Previous studies have shown the following:

<u>Bilirubin Elevations</u>: Bilirubin elevations of greater than 1.5x the upper limit of normal were observed in <1% of subjects treated with LDV/SOF for 12 weeks.

<u>Lipase Elevations</u>: Transient, asymptomatic lipase elevations of greater than 3x the upper limit of normal were observed in 2% of subjects treated with LDV/SOF12 weeks.

<u>Creatine Kinase</u>: Creatine kinase was not assessed in Phase 3 trials of LDV/SOF FDC. Isolated, asymptomatic creatine kinase elevations (Grade 3 or 4) have been previously reported in subjects treated with sofosbuvir in combination with ribavirin or peginterferon/ribavirin in other clinical trials.

There are known drug interactions with LDV/SOF and other prescription and non-prescription medications. A comprehensive list of medications will be obtained from participants including over-the-counter medications (i.e. antacids, St. John's wort) and compared to the package insert to assess any contraindication to participation or necessary alterations in dosing schedules with use of the study medication.

There is a risk of Hepatitis B virus (HBV) reactivation while receiving treatment for HCV in patients who are co-infected with HBV and HCV. HBV reactivation has been reported, in some cases resulting in fulminant hepatitis, hepatic failure and death.

Phlebotomy or starting an IV may lead to excessive bleeding, discomfort, feelings of dizziness or faintness, and/or bruising, swelling and/or infection.

Disclosure of HIV status may cause worry, sadness or depression. Disclosure of HIV-positive status has been associated with depression, suicidal ideation, and denial as well as social isolation. Trained counselors will be available to help participants deal with these feelings.

Participation in clinical research includes the risks of confidentiality loss.

Participants will be asked to provide personal/protected health information (PHI). All attempts will be made to keep PHI confidential within the limits of the law. However, there is a chance that unauthorized persons will see PHI. All paper records will be kept in a locked file cabinet or maintained in a locked room at Magee. Electronic files will be password protected. Only people who are involved in the conduct, oversight, monitoring, or auditing of this study will be allowed access to the PHI that is collected. Any publications from this study will not use information that will identify participants

by name. Organizations that may inspect and/or copy research records maintained at the participating sites for quality assurance and data analysis include groups such as the study sponsor, the National Institute of Health (NIH) or its designee and the US Food and Drug Administration (FDA).

Fetus/infant:

There is not data on use of this medication during pregnancy so the effects on a fetus/unborn child are not known. The study medication may cross the placenta and get to the fetus/unborn infant exposing the fetus to the study medication. It is unknown whether a fetus/unborn infant whose mother took LDV/SOF during pregnancy will develop normally or have side effects.

Obtaining blood samples from the infant may cause them to cry, cause bleeding, bruising or a clot and may be distressful to watch the infant have their blood drawn.

13.3.2 Benefits

This study is a safety study of use of LDV/SOF in pregnancy and as such the study medication may not treat HCV in pregnancy as it may not be the correct dose. Participants and others may benefit in the future from information learned from this study. Specifically, information learned in this study may lead evidence based guidance for the treatment of chronic hepatitis C infection in pregnancy and prevention of perinatal HCV transmission.

13.4 Informed Consent Process

Written informed consent will be obtained from each study participant prior to performing study procedures. In obtaining and documenting informed consent, the study investigators will comply with applicable local and US regulatory requirements and will adhere to GCP and to the ethical principles that have their origin in the Declaration of Helsinki. Participants may be provided with a copy of the informed consent form if they chose.

The informed consent process will cover all elements of informed consent required by research regulations. In addition, the process specifically will address the following topics of importance to this study:

- The unknown safety and proven efficacy of the study medications
- The importance of daily adherence to the study medication
- The importance of adherence to the study visit and procedures schedule
- The potential medical risks of study participation (and what to do if such risks are experienced)
- The potential social harms associated with study participation (and what to do if such harms are experienced)

- The benefits of study participation
- The distinction between research and clinical care
- The right to withdraw from the study at any time

13.5 Participant Confidentiality

All study procedures will be conducted in private, and every effort will be made to protect participant privacy and confidentiality to the extent possible. All study-related information will be stored securely at the clinical research unit. All participant information will be stored in locked areas with access limited to the clinical study staff. All laboratory specimens, study data collection, and administrative forms will be identified by coded number (PTID) only to maintain participant confidentiality. All records that contain names or other personal identifiers, such as locator forms and informed consent forms, will be stored separately from study records identified by code number following completion of the study. All local databases will be secured with password protected access systems. Forms, lists, logbooks, appointment books, and any other listings that link participants' ID numbers (PTID) to identifying information will be stored in a separate, locked area with limited access. Participants' study information will not be released without their written permission, except as necessary for review, monitoring, and/or auditing by the following:

- Representatives of the US Federal Government, including the US FDA, the US OHRP and other local and US regulatory authorities
- Representatives of Gilead Sciences
- Study staff
- University of Pittsburgh IRB
- University of Pittsburgh Research Conduct and Compliance office

13.6 Special Populations

13.6.1 Pregnant Women

Pregnant women will be offered enrollment in this study in accordance with guidelines set forth in the US 45 CFR 46.

13.6.2 Children

Infant procedures will begin at the time of delivery if delivered at Magee-Womens Hospital or at iV1 visit if infant delivered outside of Magee-Womens Hospital in accordance with guidelines set forth in the US 45 CFR 46 and DAIDS policy (http://www.niaid.nih.gov/LabsAndResources/resources/DAIDSClinRsrch/Documents/enrollingchildrenrequirements.pdf).

13.7 Compensation

Pending IRB approval, participants will be compensated for time and effort in this study, and/or be reimbursed for travel to study visits and time away from work. Reimbursement amounts will be specified in the informed consent form. Compensation may include parking/bus passes and additional incentives for making study appointments within the window period. If needed and desired, compensation for overnight stay during the PK-1, PK-2 and PK-3 visits will be provided to participants traveling over 2 hours one-way to Magee-Womens Hospital for their study visits.

13.8 Communicable Disease Reporting

Study staff will comply with local requirements to report communicable diseases including HIV-1 identified among study participants to health authorities. Participants will be made aware of reporting requirements during the informed consent process.

13.9 Access to HIV-related Care

13.9.1 HIV Counseling and Testing

HIV testing will be performed at Screening. HIV test-related counseling will be provided to all potential study participants who consent to undergo HIV-1 screening to determine eligibility for participation. Counseling will be provided in accordance with standard HIV counseling policies and methods. Participants are expected to have HIV screening as part of routine prenatal care at 28 weeks' gestation, and if clinically indicated during the study.

13.9.2 Care for Participants Identified as HIV-Positive

<u>Identified as HIV-Positive Prior to Enrollment</u>
An individual who has been identified as infected with HIV-1 will not be eligible to participate and will be referred to the Pittsburgh AIDS Center for Treatment (PACT).

<u>Identified as HIV-Positive While on Study Medication</u>
The participant will continue with the study medication and study procedures, and will be immediately referred to PACT.

13.10 Study Discontinuation

This study may be discontinued at any time by Gilead Sciences, the US FDA, the OHRP, other government or regulatory authorities, or the University of Pittsburgh IRB.

14 PUBLICATION POLICY

The University of Pittsburgh study investigators will be responsible for publication of the results of this study. The manuscript draft will be sent to Gilead Sciences 30 days prior to submission for their review and approval.

15 APPENDICES

APPENDIX I: SCHEDULE OF STUDY VISITS AND EVALUATIONS (Mother)

	SCR (V1)	ENR (V2)	PK1, PK2, PK3 (V3, V4, V5)	End of Treatment (V6)	Delivery**	Post treatment (V7)
**ADMINISTRATIVE AND REGULATORY**						
Informed consent(s)	X					
Assess informed consent comprehension	X					
Assignment of PTID	X					
Locator information	X	X	X	X	X	X
Demographic information	X					
Eligibility assessment	X					
Eligibility confirmation		X				
Reimbursement	X	X	X	X	X	X
Record/ update AEs			X	X	X	X
Schedule next visit	X	X	X		X	-
**BEHAVIORAL**						
HIV pre- and post- test counseling	X					
Protocol adherence counseling		X	X	X	X	X
Medication use/adherence counseling		X	X		*	
In depth interview		X		X		
**CLINICAL**						
Medical history	X	X	X	X	X	X
Concomitant medications	X	X	X	X	X	X
Document pre-existing conditions	X					
Physical examination (full or modified)	X	X	X	X	X	X
Assessment of fetal heart tones	X	X	X	X		
Provide available test results	X	X	X	X	X	X
Collect Pregnancy Outcomes					X	X
**LABORATORY**						
HCV RNA viral load	X	X	X (PK1&2)	X	X	X
HCV Genotype	X					
HCV Resistance testing			*(PK2 only)	*	*	*
HBV Testing	X					
CBC	X		X (PK1&2)			X
Creatinine	X		X (PK1&2)			X
Hepatic Function Panel	X		X (PK1&2)			X
Lipase	X		X (PK1&2)			X
Creatinine kinase	X		X (PK1&2)			X
HIV serology	X	*	*	*	*	*
PT/INR	X		X (PK1&2)			X
SOF PK			X		^	
LDV PK			X		^	
Protein Binding Assay			X		^	
**STUDY MEDICATION**						
Provision of Study Medication		X	X			
Collect Remaining Study Medication				X		

*If clinically indicated

**May be performed during hospital admission for participants who deliver at Magee or applicable procedures conducted by phone for participants who deliver at outside locations. For all participants, a chart review of the delivery records will be done as part of the Delivery Visit

^ If still on study medication at the time of delivery

APPENDIX II: SCHEDULE OF STUDY VISITS AND EVALUATIONS (Infant)

	Delivery***	iV1 (Month 1-3)	iV2 (Month 6)	iV3 (Month 12)
**ADMINISTRATIVE AND REGULATORY**				
Assignment of PTID	X			
Locator information	X	X	X	X
Demographic information	X			
Reimbursement	X	X	X	X
Record/update AEs		X	X	X
Schedule next visit	X	X	X	
**CLINICAL**				
Medical history **	X	X	X	X
Concomitant medications**	X	X	X	X
Document pre-existing conditions**	X	X		
Physical examination **	X	X	X	X
Collect growth parameters **	X	X	X	X
Collect Baseline Information**	X			
Provide available test results		X	X	X
Neurodevelopmental Exam			X	X
**LABORATORY**				
HCV RNA viral load	X	X	X	X*
HCV Resistance testing	*	*	*	*
Protein Binding Assay	^			
LDV PK	^			
SOF PK	^			

*If clinically indicated
**May be obtained from medical record as applicable
*** May be performed during hospital admission for infants born at Magee or applicable procedures conducted by phone with mother for infants who deliver at outside locations. For all participants, a chart review of the delivery records will be done as part of the Delivery Visit.
^ If mother on study drug at the time of delivery and delivers at Magee-Womens Hospital

Reference List

1. Hepatitis C Factsheet. World Health Organization. http://www.who.int/mediacentre/factsheets/fs164/en/. Accessed on March 19, 2015.
2. Guidelines for Viral Hepatitis Surveillances and Case Management. Centers for Disease Control and Prevention. http://www.cdc.gov/hepatitis/Statistics/SurveillanceGuidelines.htm#hepc. Accessed on March 19, 2015.
3. American College of Obstetricans and Gynecologist: ACOG Practice Bulletin No. 86: Viral hepatitis in pregnancy. Obstet Gynecol 2007; 110: 941-955.
4. Charlton M. Hepatitis C infection in liver transplantation. Am J Transplant 2001;1:197-203.
5. Connell LE, Salihu HM, Salemi JL, August EM, Weldeselasse H, Mbah AK. Maternal hepatitis B and hepatitis C carrier status and perinatal outcomes. Liver Int 2011;31:1163-70.
6. Reddick KL, Jhaveri R, Gandhi M, James AH, Swamy GK. Pregnancy outcomes associated with viral hepatitis. J Viral Hepat 2011;18:e394-8.
7. Berkley EM, Leslie KK, Arora S, Qualls C, Dunkelberg JC. Chronic hepatitis C in pregnancy. Obstet Gynecol 2008;112:304-10.
8. Pergam SA, Wang CC, Gardella CM, Sandison TG, Phipps WT, Hawes SE. Pregnancy complications associated with hepatitis C: data from a 2003-2005 Washington state birth cohort. Am J Obstet Gynecol 2008;199:38 e1-9.
9. Paternoster DM, Santarossa C, Grella P, et al. Viral load in HCV RNA-positive pregnant women. Am J Gastroenterol 2001;96:2751-4.
10. Gervais A, Bacq Y, Bernuau J, et al. Decrease in serum ALT and increase in serum HCV RNA during pregnancy in women with chronic hepatitis C. J Hepatol 2000;32:293-9.
11. Salemi JL, Whiteman VE, August EM, Chandler K, Mbah AK, Salihu HM. Maternal hepatitis B and hepatitis C infection and neonatal neurological outcomes. J Viral Hepat 2014;21:e144-53.
12. Marcellin P, Boyer N, Gervais A, et al. Long-term histologic improvement and loss of detectable intrahepatic HCV RNA in patients with chronic hepatitis C and sustained response to interferon-alpha therapy. Ann Intern Med 1997;127:875-81.
13. Coppola N, De Pascalis S, Pisaturo M, et al. Sustained virological response to antiviral treatment in chronic hepatitis C patients may be predictable by HCV-RNA clearance in peripheral blood mononuclear cells. J Clin Virol 2013;58:748-50.
14. Garcia-Bengoechea M, Basaras M, Barrio J, et al. Late disappearance of hepatitis C virus RNA from peripheral blood mononuclear cells in patients with chronic hepatitis C in sustained response after alpha-interferon therapy. Am J Gastroenterol 1999;94:1902-5.
15. Manns MP, Pockros PJ, Norkrans G, et al. Long-term clearance of hepatitis C virus following interferon alpha-2b or peginterferon alpha-2b, alone or in combination with ribavirin. J Viral Hepat 2013;20:524-9.
16. Swain MG, Lai MY, Shiffman ML, et al. A sustained virologic response is durable in patients with chronic hepatitis C treated with peginterferon alfa-2a and ribavirin. Gastroenterology 2010;139:1593-601.
17. Poynard T, McHutchison J, Manns M, et al. Impact of pegylated interferon alfa-2b and ribavirin on liver fibrosis in patients with chronic hepatitis C. Gastroenterology 2002;122:1303-13.
18. Morgan RL, Baack B, Smith BD, Yartel A, Pitasi M, Falck-Ytter Y. Eradication of hepatitis C virus infection and the development of hepatocellular carcinoma: a meta-analysis of observational studies. Ann Intern Med 2013;158:329-37.
19. van der Meer AJ, Veldt BJ, Feld JJ, et al. Association between sustained virological response and all-cause mortality among patients with chronic hepatitis C and advanced hepatic fibrosis. Jama 2012;308:2584-93.
20. Fabrizi F, Dixit V, Messa P. Antiviral therapy of symptomatic HCV-associated mixed cryoglobulinemia: meta-analysis of clinical studies. J Med Virol 2013;85:1019-27.
21. Takahashi K, Nishida N, Kawabata H, Haga H, Chiba T. Regression of Hodgkin lymphoma in response to antiviral therapy for hepatitis C virus infection. Intern Med 2012;51:2745-7.
22. Gisbert JP, Garcia-Buey L, Pajares JM, Moreno-Otero R. Systematic review: regression of lymphoproliferative disorders after treatment for hepatitis C infection. Aliment Pharmacol Ther 2005;21:653-62.

23. Recommendations for Testing, Managing and Treating Hepatitis C. Infectious Diseases Society of America and American Association for the Study of Liver Diseases. Accessed at: http://www.hcvguidelines.org/fullreport on August 10,2016.
24. Ghany MG, Nelson DR, Strader DB, Thomas DL, Seeff LB. An update on treatment of genotype 1 chronic hepatitis C virus infection: 2011 practice guideline by the American Association for the Study of Liver Diseases. Hepatology 2011;54:1433-44.
25. Manns MP, McHutchison JG, Gordon SC, et al. Peginterferon alfa-2b plus ribavirin compared with interferon alfa-2b plus ribavirin for initial treatment of chronic hepatitis C: a randomised trial. Lancet 2001;358:958-65.
26. Fried MW, Shiffman ML, Reddy KR, et al. Peginterferon alfa-2a plus ribavirin for chronic hepatitis C virus infection. N Engl J Med 2002;347:975-82.
27. Kowdley KV, Gordon SC, Reddy KR, et al. Ledipasvir and sofosbuvir for 8 or 12 weeks for chronic HCV without cirrhosis. N Engl J Med 2014;370:1879-88.
28. Afdhal N, Reddy KR, Nelson DR, et al. Ledipasvir and sofosbuvir for previously treated HCV genotype 1 infection. N Engl J Med 2014;370:1483-93.
29. Afdhal N, Zeuzem S, Kwo P, et al. Ledipasvir and sofosbuvir for untreated HCV genotype 1 infection. N Engl J Med 2014;370:1889-98.
30. Kohli A, Kapoor R, Sims Z, et al. Ledipasvir and sofosbuvir for hepatitis C genotype 4: a proof-of-concept, single-centre, open-label phase 2a cohort study. Lancet Infect Dis 2015;15:1049-54.
31. Abergel A, Metivier S, Samuel D, et al. Ledipasvir plus sofosbuvir for 12 weeks in patients with hepatitis C genotype 4 infection. Hepatology 2016;64:1049-56.
32. Abergel A, Asselah T, Metivier S, et al. Ledipasvir-sofosbuvir in patients with hepatitis C virus genotype 5 infection: an open-label, multicentre, single-arm, phase 2 study. Lancet Infect Dis 2016;16:459-64.
33. Gane EJ, Hyland RH, An D, et al. Efficacy of ledipasvir and sofosbuvir, with or without ribavirin, for 12 weeks in patients with HCV genotype 3 or 6 infection. Gastroenterology 2015;149:1454-61 e1.
34. Benova L, Mohamoud YA, Calvert C, Abu-Raddad LJ. Vertical transmission of hepatitis C virus: systematic review and meta-analysis. Clin Infect Dis 2014;59:765-73.
35. Nachega JB, Uthman OA, Anderson J, et al. Adherence to antiretroviral therapy during and after pregnancy in low-income, middle-income, and high-income countries: a systematic review and meta-analysis. Aids 2012;26:2039-52.
36. Ngarina M, Tarimo EA, Naburi H, et al. Women's preferences regarding infant or maternal antiretroviral prophylaxis for prevention of mother-to-child transmission of HIV during breastfeeding and their views on Option B+ in Dar es Salaam, Tanzania. PLoS One 2014;9:e85310.
37. Ngarina M, Popenoe R, Kilewo C, Biberfeld G, Ekstrom AM. Reasons for poor adherence to antiretroviral therapy postnatally in HIV-1 infected women treated for their own health: experiences from the Mitra Plus study in Tanzania. BMC Public Health 2013;13:450.
38. Harvoni ® [package insert]. Foster City, CA:Gilead Sciences, Inc. March 2015.
39. Hebner C, Lee Y-J, Han B, et al. In vitro pan-genotypic and combination activity of sofosbuvir (GS-7977) in stable replicon cell lines [abstract 1875]. Hepatology. 2012;56(4 Suppl):1066A.
40. Keating GM. Ledipasvir/Sofosbuvir: a review of its use in chronic hepatitis C. Drugs 2015;75:675-85.
41. Kirby BJ, Symonds WT, Kearney BP, Mathias AA. Pharmacokinetic, Pharmacodynamic, and Drug-Interaction Profile of the Hepatitis C Virus NS5B Polymerase Inhibitor Sofosbuvir. Clin Pharmacokinet 2015;54:677-90.
42. German P, Moorehead L, Pang P, Vimal M, Mathias A. Lack of a clinically important pharmacokinetic interaction between sofosbuvir or ledipasvir and hormonal oral contraceptives norgestimate/ethinyl estradiol in HCV-uninfected female subjects. J Clin Pharmacol 2014;54:1290-8.
